# Self‐Healing COCu‐Tac Hydrogel Enhances iNSCs Transplantation for Spinal Cord Injury by Promoting Mitophagy via the FKBP52/AKT Pathway

**DOI:** 10.1002/advs.202407757

**Published:** 2024-11-25

**Authors:** Zhenming Tian, Han‐Jian Hu, Chun Cheung Chan, Tian Hu, Chaoyang Cai, Hong Li, Limin Rong, Gang‐Biao Jiang, Bin Liu

**Affiliations:** ^1^ Department of Spine Surgery The Third Affiliated Hospital of Sun Yat‐Sen University Guangzhou 510630 China; ^2^ Guangdong Provincial Center for Quality Control of Minimally Invasive Spine Surgery Guangzhou 510630 China; ^3^ Guangdong Provincial Center for Engineering and Technology Research of Minimally Invasive Spine Surgery Guangzhou 510630 China; ^4^ Key Laboratory for Biobased Materials and Energy of Ministry of Education College of Materials and Energy South China Agricultural University Guangzhou 510642 China

**Keywords:** FKBP52, induced neural stem cells, mitophagy, self‐healing hydrogel, spinal cord injury

## Abstract

In the realm of neural regeneration post‐spinal cord injury, hydrogel scaffolds carrying induced neural stem cells (iNSCs) have demonstrated significant potential. However, challenges such as graft rejection and dysfunction caused by mitochondrial damage persist after transplantation, presenting formidable barriers. Tacrolimus, known for its dual role as an immunosuppressant and promoter of neural regeneration, holds the potential for enhancing iNSC transplantation. However, systemic administration of tacrolimus often comes with severe side effects. This study pioneers the development of a self‐healing hydrogel with sustained‐release tacrolimus (COCu‐Tac), tailored specifically for iNSC transplantation after spinal cord injury. This research reveals that the sustained release of tacrolimus enhances axonal growth and improves mitochondrial quality control in iNSCs and neurons. Further analysis shows that tacrolimus targets FKBP52 rather than FKBP51, enhancing mitophagy via the FKBP52/AKT pathway. This advanced system demonstrates significant efficacy in promoting neural regeneration and restoring motor function following spinal cord injury.

## Graphical abstract

A novel hydrogel‐based transplant system (COCu‐Tac) for induced neural stem cell (iNSCs) therapy in spinal cord injury is introduced. This system offers self‐healing properties, sustained tacrolimus release, and reduces immune rejection without systemic immunosuppression. It promotes iNSC survival, axonal growth, and neural regeneration through mitophagy enhancement via the FKBP52/AKT pathway, demonstrating potential for broader tissue regeneration applications.

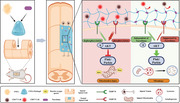

## 1 Introduction

Spinal cord injury (SCI) presents a significant clinical challenge due to the limited regenerative capacity of the central nervous system.^[^ ^1^, ^2^ ^]^ Scaffolds loaded with neural stem cells (NSCs) have emerged as a promising therapeutic approach.^[^ ^3^ ^]^ Induced pluripotent stem cells(iPSCs)‐derived neural stem cells (iNSCs), known for their potential for autologous transplantation and unlimited self‐renewal, offer a robust solution for regenerating damaged neural pathways in SCI.^[^ ^4^, ^5^, ^6^ ^]^ However, graft rejection and energy metabolism disorders are major obstacles to cell transplantation after SCI.^[^ ^7^ ^]^

To prevent graft rejection, immunosuppressive drugs like tacrolimus (alias FK506), which inhibit T‐cell activation, have been clinically utilized.^[^ ^8^ ^]^ The effectiveness of tacrolimus as an immunosuppressant has been validated in various transplantation scenarios. It also shows promise in promoting neural regeneration by enhancing axonal growth.^[^ ^9^ ^]^ However, the systemic administration of tacrolimus is often associated with severe side effects.^[^ ^10^, ^11^ ^]^ In this study, we have modified our previously reported self‐healing hydrogel to incorporate controlled tacrolimus release (COCu‐Tac), providing local protection against graft rejection in iNSCs transplantation.^[^ ^12^ ^]^ Despite these advances, research on the influence of tacrolimus on energy metabolism in transplantation is currently lacking.

Disturbances in energy metabolism caused by mitochondrial damage can lead to oxidative stress, exacerbating secondary injuries to spinal cord tissues.^[^ ^13^ ^]^ As a key component of mitochondrial quality control (MQC), mitophagy involves the selective degradation of damaged mitochondria. When energy metabolism disorder occur, the Pink1/Parkin pathway mediates mitophagy to eliminate the dysfunctional mitochondria to restore MQC.^[^ ^14^ ^]^ This process is essential for maintaining cellular health and enhancing the success of cell transplantation strategies for SCI repair. Recent studies have highlighted the role of mitophagy in enhancing the survival rates of transplanted cells and organs by aiding their adaptation to stress conditions such as ischemia‐reperfusion injury (IRI),^[^ ^15^, ^16^ ^]^ which closely mirrors the secondary damage observed in SCI.^[^ ^17^ ^]^

**Scheme 1:**
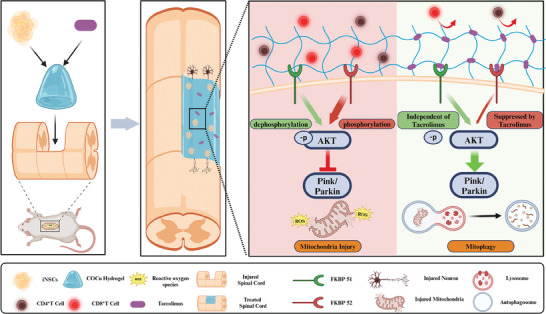
Schematic representation of the COCu‐Tac‐iNSC hydrogel promoting mitophagy to repair spinal cord injury by targeting FKBP52.

Recent studies have found that FK506 binding proteins (FKBPs) play a regulatory role in mitophagy. FKBP51(encoded by the *FKBP5* gene) and FKBP52 (encoded by the *FKBP4* gene) are members of the immunophilin family of FKBPs, sharing a 70% sequence similarity and exhibiting antagonistic functions in various processes. Tacrolimus acts on the FK1 domain of FKBP51/52 to inhibit their PPIase activity.^[^ ^18^ ^]^ Previous studies have indicated that FKBP51 colocalizes with Parkin in the mitochondrial network and promotes AKT dephosphorylation and mitophagy.^[^ ^19^, ^20^ ^]^ FKBP52 is also recognized as a mitochondrial‐related receptor and is involved in promoting AKT phosphorylation.^[^ ^21^, ^22^, ^23^ ^]^ Our research revealed that tacrolimus activates mitophagy in iNSCs by targeting FKBP52 to inhibit AKT phosphorylation. In contrast, FKBP51's promotion of AKT dephosphorylation and mitophagy is independent of its PPIase activity and not influenced by tacrolimus. The COCu‐Tac hydrogel demonstrates controlled release of tacrolimus and selectively targets FKBP52 over FKBP51 to inhibit AKT phosphorylation in iNSCs, thereby promoting mitophagy and enhancing MQC (**Scheme** **1**).

In summary, we introduced a novel transplant system (COCu‐Tac) for iNSCs therapy following SCI. Our study found that tacrolimus promotes the survival of transplanted iNSCs through its dual functions of immunosuppression and mitochondrial protection. This protective action is mediated by tacrolimus targeting FKBP52 to inhibit AKT phosphorylation and enhance mitophagy. This advanced iNSCs transplantation therapy significantly contributed to neural regeneration and functional recovery in animal models.

## 2 Results

### 2.1 Fabrication and Characterization of COCu Hydrogel

We synthesized aldehyde‐modified chitosan oligosaccharide (OCS) through an amidation reaction between the amino group of chitosan and the carboxyl group of 4‐formylbenzoic acid (4‐FA) as illustrated in Figure S1A, (Supporting Information). Fourier‐transform infrared spectroscopy (FTIR) confirmed the reaction; the OCS spectrum displayed a C≐O absorption peak at 1697 cm^−1^ corresponding to 4‐FA, a new C≐O peak at 1630 cm^−1^, and a narrower amino group peak at 3330 cm^−1^, indicating successful synthesis (Figure S1B,C, Supporting Information). Additionally, 1H NMR analysis identified five new peaks in OCS, verifying the successful synthesis of aldehyde‐modified chitosan oligosaccharides.

**Figure 1:**
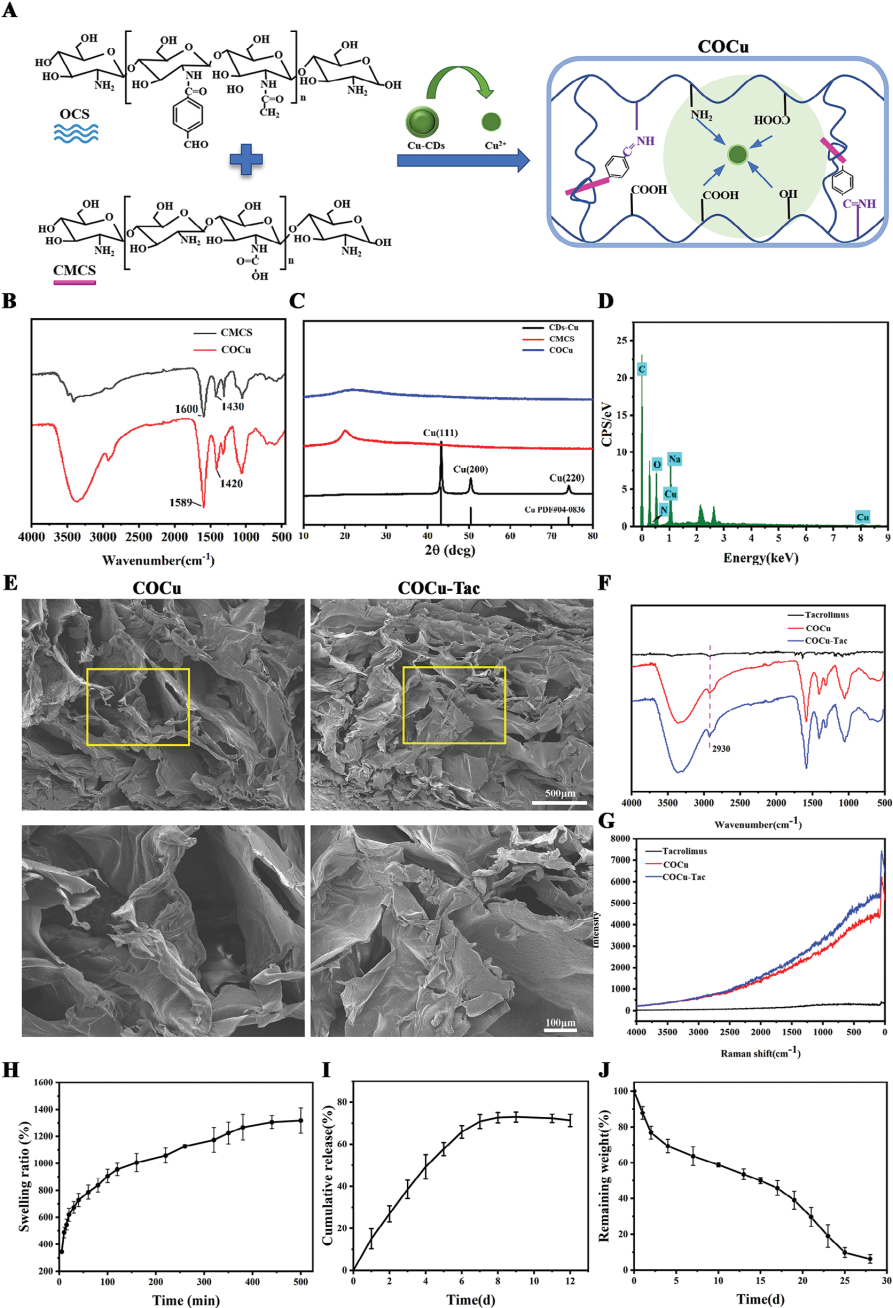
Analysis of hydrogel formation mechanism. A) Schematic diagram illustrating the synthesis of COCu hydrogel; B) Infrared spectra comparison between CMCS and COCu hydrogel; C) XRD patterns for CD‐Cus, CMCS, and COCu hydrogel; D) EDX analysis of COCu hydrogel; E) SEM images of COCu and COCu‐Tac hydrogels, Scale bar, 500 µm for original pictures and 100 µm for enlarged pictures; F,G) FTIR and Raman spectroscopy of Tacrolimus, COCu Hydrogel, and COCu‐Tac Hydrogel; H) Swelling curve of COCu‐Tac Hydrogel; I) Tacrolimus release curve of COCu‐Tac Hydrogel; J) In vitro degradation curves of COCu‐Tac Hydrogel.

The COCu hydrogel is primarily formed through the Schiff base reaction between the aldehyde groups of OCS and the amino groups of carboxymethyl chitosan (CMCS), complemented by the coordination reaction of Cu^2+^ derived from copper‐doped carbon dots (Cu‐CDs) with the amino and carboxyl groups of CMCS (**Figure** **1**A). FTIR analysis of the COCu spectrum revealed coordination between the amino groups in CMCS and copper ions, as evidenced by the shift in the N─H deformation vibration peak. Additionally, a shift in the O─H deformation vibration peak of carboxyl groups confirmed the coordination reaction with Cu^2+^ (Figure 1B). X‐ray powder diffraction (XRD) analysis further validated the role of Cu‐CDs in the synthesis of the COCu hydrogel, showing three diffraction peaks corresponding to face‐centered cubic copper (JCPDS#04‐0836), with no peak indicative of oxidized copper crystals. These peaks were absent in the COCu hydrogel, indicating a complete transformation of Cu‐CDs into Cu^2+^ (Figure 1C). Scanning electron microscopy (SEM) demonstrated a smooth and particle‐free surface of the hydrogel, and energy‐dispersive X‐ray spectroscopy (EDX) confirmed the presence of copper elements, demonstrating successful incorporation of Cu‐CDs in the form of Cu^2+^ (Figure 1D,E).

### 2.2 Characterization of COCu‐Tac Hydrogel

The tacrolimus‐loaded COCu hydrogel (COCu‐Tac) was synthesized and COCu‐Tac hydrogel with 20 µm tacrolimus was used for the material study. SEM revealed the porous structure of the COCu hydrogel, which is highly conducive to the proliferation and differentiation of neural cells. This structure also facilitates the transport of nutrients and metabolic waste, providing an ideal artificial extracellular matrix (ECM) for neural regeneration. The COCu‐Tac hydrogel displays a relatively rough surface, indicative of the successful incorporation of tacrolimus (Figure 1E). FTIR and Raman spectroscopy further confirmed the integration of tacrolimus (Figure 1F,G). The hydrogel exhibited an equilibrium swelling ratio of ≈1317%, indicating that the COCu hydrogel possesses excellent regenerative capacity after dehydration^[^ ^12^ ^]^ (Figure 1H). The hydrogel demonstrated sustained tacrolimus release in vitro, reaching equilibrium on the eighth day, which aligns with the timeline of pathological changes observed in SCI^[^ ^24^ ^]^ (Figure 1I). Ideally, a hydrogel for SCI repair should degrade gradually alongside the regeneration of neural tissue. The COCu‐Tac hydrogel achieved slow and relatively uniform degradation within 28 days, a rate well‐suited for SCI repair (Figure 1J).

### 2.3 Mechanical Characteristics of COCu Hydrogel

The mechanical properties of the hydrogel within the matrix environment are essential for influencing cell functions and differentiation. We analyzed the storage modulus (G’) and loss modulus (G″) of the hydrogel at different frequencies. The maximum storage modulus was 2386 Pa, and the modulus difference was 1218 Pa, aligning with reported values for spinal cord tissues (100–3000 Pa) (Figure S2A, Supporting Information).^[^ ^25^ ^]^ The hydrogel also demonstrated the ability to withstand substantial pressure while maintaining its gel state (Figure S2B, Supporting Information). The viscosity of the hydrogel increased with shear rates up to 0.68 s^−1^ and decreased beyond this point (Figure S2C, Supporting Information).

Given the back's high activity, the material must continuously resist external force deformations.^[^ ^26^ ^]^ We conducted cyclic breakage‐healing tests at alternating low (1%) and high (150%) strains to assess sustained self‐healing. The hydrogel maintained a stable gel state at low strains and exhibited rapid recovery after high strains (Figure S2D, Supporting Information).

To demonstrate the hydrogel's applicability in SCI defects, we affixed it to a glass slide with spinal cord tissue. The hydrogel securely adhered to the spinal tissue, preventing displacement (Figure S2E, Supporting Information). Additionally, when two pieces of COCu hydrogel were joined, they fused within 2 min (Figure S2F,G, Supporting Information). An experiment placing the hydrogel over glass beads in an empty beaker confirmed its capability to fill gaps effectively and secure the beads at the bottom (Figure S2H, Supporting Information). These experiments validate the hydrogel's robust tissue adhesion, self‐healing, and self‐adapting properties, making it suitable for various SCI defect applications.

### 2.4 Induction and Identification of iNSCs

iNSCs were derived from iPSCs and formed neural spheres in ultra‐low attachment culture dishes (Figure S3A,B, Supporting Information). Both iPSCs and iNSCs were characterized through immunofluorescence and RT‐PCR. The iNSCs exhibited low expression of pluripotency markers (OCT4 and NANOG) but showed higher levels of NSCs‐specific markers (Nestin and Pax6) compared to iPSCs (Figure S3C,D, Supporting Information). The in vitro differentiation of iNSCs into neurons, astrocytes, and oligodendrocytes within 7 days further demonstrated their potential for SCI repair (Figure S3E, Supporting Information).

### 2.5 Biocompatibility of COCu‐Tac Hydrogel

**Figure 2:**
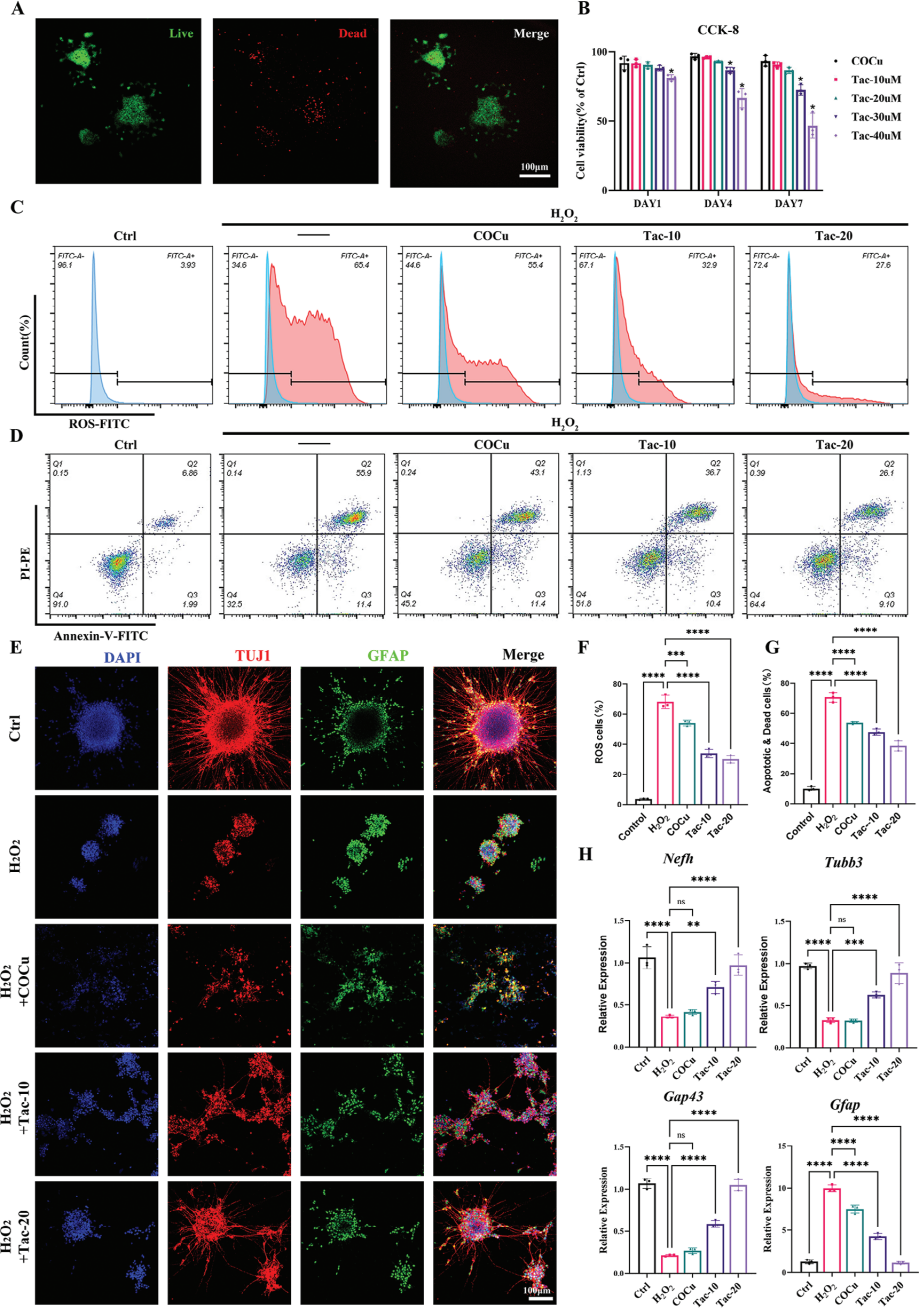
COCu‐Tac hydrogel protects iNSCs under oxidative stress. A) Live‐dead staining of iNSC on COCu hydrogels; B) CCK‐8 experiment of iNSC on COCu‐Tac hydrogels at different concentrations (*n* = 3); C) Flow cytometry plot of ROS positive iNSC; D) Flow cytometry plot of apoptotic and dead iNSC on COCu‐Tac hydrogels at different concentrations; E) The immunofluorescence staining of differentiated iNSC, neurons and astrocytes were labeled with Tuj1 and GFAP respectively, Scale bar, 100 µm; F) Quantitative analysis of the ratio of ROS positive iNSCs in (C) (*n* = 3); G) Quantitative analysis of the ratio of apoptotic and dead iNSCs in (D) (*n* = 3); H) Expression levels of neuron and astrocyte markers in differentiated iNSCs (*n* = 3), ^*^ *p* < 0.05, ^***^ *p* < 0.001, ns means no significance.

High tacrolimus loading can facilitate prolonged drug release and support the survival of transplanted tissue.^[^ ^27^ ^]^ However, excessively high concentrations of tacrolimus may inhibit cell proliferation. To determine the optimal drug‐loading concentration for COCu‐Tac, we evaluated the survival and proliferation of iNSCs using Live‐Dead and CCK‐8 assays across various concentrations of COCu‐Tac. After 7 days, the majority of cells remained viable on the COCu hydrogel (**Figure** **2**A). CCK‐8 assays revealed that iNSCs maintained similar proliferation rates on both COCu and COCu‐Tac with 10 µm tacrolimus (Tac‐10) compared to a control plate. On COCu‐Tac with 20 µm tacrolimus (Tac‐20), cells retained ≈88% of their proliferative capacity by day 7. However, this rate declined to 67% and 48% as tacrolimus concentrations increased to 30 and 40 µm, respectively (Figure 2B). Therefore, the tacrolimus concentration in COCu‐Tac should not exceed 20 µm, aligning with findings from previous studies.^[^ ^28^, ^29^, ^30^ ^]^

### 2.6 Survival and Differentiation of iNSCs Under Oxidative Stress on COCu‐Tac

The substantial increase in reactive oxygen species (ROS), known to activate various cell death mechanisms during the acute phase of SCI, underscores a connection between neuronal loss and oxidative damage.^[^ ^31^ ^]^ In response, we evaluated the protective effects of COCu‐Tac on iNSCs in an oxidative stress environment. Notably, the COCu hydrogel alone exhibited modest antioxidative effects, and the sustained release of tacrolimus further reduced cellular ROS levels (Figure 2C,E).

Additionally, we investigated iNSCs apoptosis and differentiation under oxidative stress. After H_2_O_2_‐induced damage, iNSCs showed increased apoptosis and a tendency to differentiate into astrocytes. As the tacrolimus concentration in the COCu hydrogel increased, there was a noticeable decrease in cell apoptosis (Figure 2D,F) and an enhancement in neuronal differentiation (Figure 2G,H).

### 2.7 COCu‐Tac Hydrogel Protects iNSCs Through Mitophagy

**Figure 3:**
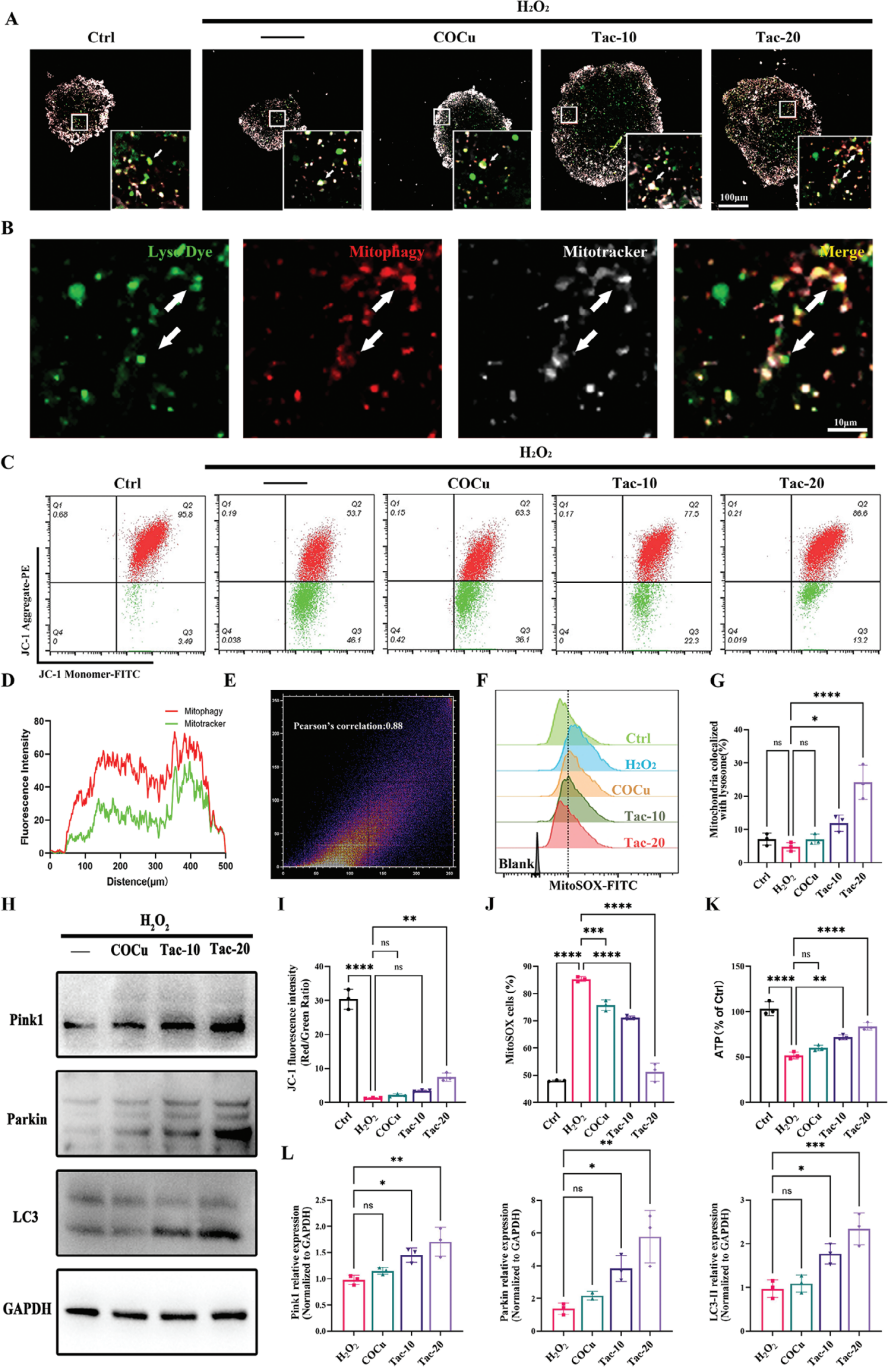
COCu‐Tac hydrogel induces iNSCs mitophagy via the PI3K/AKT pathway. A) immunofluorescence of iNSC, lysosome, mitophagy, and mitochondria were labeled with Lyso Dye, Mitophagy and, Mitotracker respectively; B) enlarged pictures of Tac‐20 group in (A), Scale bar, 100 µm for original pictures and 10 µm for enlarged pictures; C) Flow cytometry analysis of mitochondrial membrane potential (MMP) probed with JC‐1 in iNSC; D) Colocalization analysis of the fluorescence intensity of the mitophagy and mitotracker of Tac‐20 group in (A); E) Colocalization scatter plot and the Pearson's correlation coefficient of the mitophagy and mitotracker of Tac‐20 group in (A); F) Flow cytometry plot of mitochondrial ROS(MitoSOX) in iNSC; G) Quantification of the average level of colocalization between mitochondria and lysosomes in (A) (*n* = 3); H) Western blot of Pink1/Parkin/LC3 pathway; I) Quantitative analysis of the ratio of JC‐1 aggregates (referred to high MMP, PE channel)/JC‐1 monomers (referred to low MMP, FITC channel) in (C) (*n* = 3); J) Quantitative analysis of the ratio of MitoSOX positive iNSCs in (F) (*n* = 3); K) Quantitative analysis of ATP production in iNSC (*n* = 3); L) Quantitative analysis of the expression of mitophagy related proteins in (H). ^*^ *p* < 0.05,^**^ *p* < 0.01, ^***^ *p* < 0.001, ^****^ *p* < 0.0001, ns means no significance.

Mitochondria, crucial for cell survival and redox homeostasis, play a pivotal role in oxidative stress‐induced apoptosis.^[^ ^32^ ^]^ In injured cells, mitophagy is essential for maintaining MQC.^[^ ^33^ ^]^ Under oxidative stress, mitophagy in iNSCs was observed to mildly increase, a response that was further amplified by the release of tacrolimus (**Figure** **3**A,B). Although oxidative stress slightly activated iNSCs mitophagy, there was a significant decline in mitochondrial membrane potential (MMP), ATP levels, and an increase in neuronal mitochondrial ROS, suggesting that limited mitophagy could not compensate for the overall mitochondrial impairment. Despite a slight improvement in MMP and a reduction in mitochondrial ROS afforded by COCu hydrogels, there was no significant recovery in ATP levels. However, COCu‐Tac not only significantly enhanced MMP and reduced mitochondrial ROS but also restored ATP levels, indicating that tacrolimus boosts iNSCs mitophagy, thereby improving MQC and restoring mitochondrial function (Figure 3C,F,I–K). Western blot confirmed that the tacrolimus released by COCu‐Tac can activate Pink1/Parkin pathway‐mediated mitophagy (Figure 3H,L).

### 2.8 COCu‐Tac Hydrogel Promotes Mitophagy in iNSCs Through FKBP52/AKT Pathway

**Figure 4:**
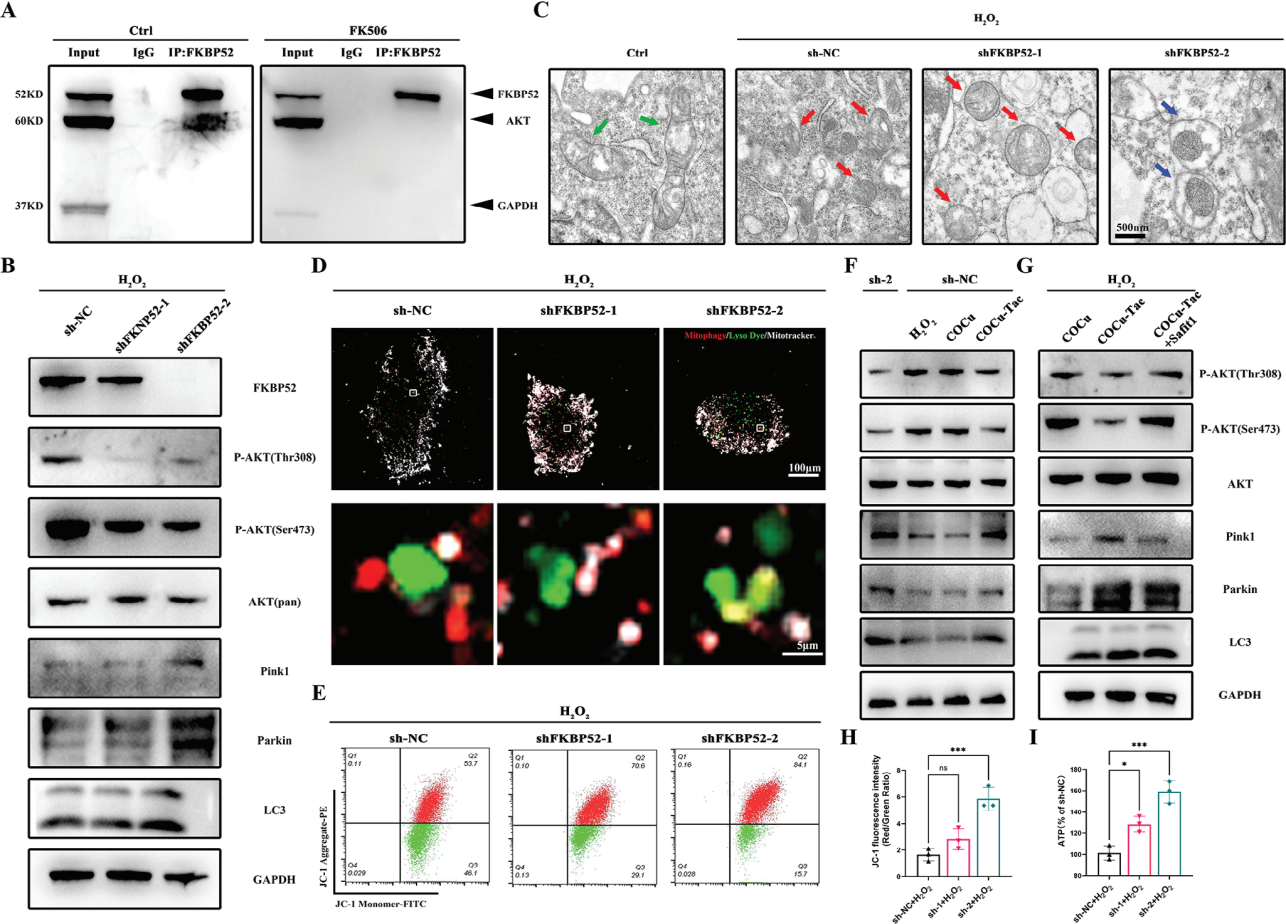
COCu‐Tac hydrogel promotes mitophagy in iNSCs through FKBP52‐mediated AKT/Pink1/Parkin pathway. A) co‐immunoprecipitation (Co‐IP) Assay of FKBP52 and AKT; B) Western blot analysis of FKBP52 and AKT/Pink1/Parkin pathway; C) Representative transmission electron microscope (TEM) images of mitochondrial morphology in iNSCs, Scale bar, 500 nm; D) immunofluorescence of iNSC mitophagy, lysosome, and mitochondria were labeled with Lyso Dye, Mitophagy and Mitotracker respectively, Scale bar, 100 µm for original pictures and 5 µm for enlarged pictures; E) Flow cytometry analysis of MMP probed with JC‐1 in iNSC; F,G) Western blot analysis of AKT/Pink1/Parkin pathway (H) Quantitative analysis of the ratio of JC‐1 aggregates/JC‐1 monomers in (E) (*n* = 3); I) Quantitative analysis of ATP production in iNSC (*n* = 3), ^*^ *p* < 0.05, ^**^ *p* < 0.01, ^***^ *p* < 0.001, ns means no significance.

Studies found that inhibiting FKBP52 suppresses AKT phosphorylation and protects against stress‐induced neuronal mitochondrial dysfunctions.^[^ ^22^, ^34^ ^]^ Using co‐immunoprecipitation (CO‐IP), we confirmed that FKBP52 interacts with AKT under physiological conditions, and this interaction can be blocked by FK506 (**Figure** **4**A). We performed FKBP52 knockout in iNSCs to investigate its impact on the AKT/Pink1/Parkin pathway. ShRNA‐mediated knockdown of FKBP52 revealed that shFKBP52‐1 caused a partial knockdown, while shFKBP52‐2 resulted in a nearly complete knockdown. As FKBP52 levels decreased, AKT phosphorylation at Thr308 and Ser473 was inhibited, the Pink1/Parkin pathway was activated, and LC3 expression increased, indicating enhanced mitophagy (Figure 4B). Transmission electron microscopy (TEM) analysis showed that control mitochondria were elongated with well‐defined cristae (green arrows), whereas damaged mitochondria appeared rounded with disrupted cristae (red arrows). FKBP52 knockdown resulted in numerous autophagosomes surrounding damaged mitochondria, suggesting increased mitophagy (blue arrows) (Figure 4C). Mitophagy probes confirmed this enhancement with FKBP52 knockdown in iNSCs (Figure 4D). JC‐1 and ATP assays demonstrated that increased mitophagy improved MMP and energy metabolism in iNSCs (Figure 4E,H,I). Western blot analysis comparing iNSCs with shFKBP52‐2 knockdown and COCu‐Tac treatment showed that COCu‐Tac mimicked the effects of FKBP52 knockdown (Figure 4F). Adding SAFit1, a specific inhibitor of FKBP51, to COCu‐Tac‐treated cells abolished the effects on AKT phosphorylation and mitophagy (Figure 4G). Since tacrolimus inhibits the FK1 domain of FKBP51, we infer that FKBP51's regulation of AKT and mitophagy is independent of its FK1 domain. Therefore, we propose that tacrolimus selectively targets FKBP52, promoting mitophagy through the FKBP52/AKT pathway.

### 2.9 COCu‐Tac Hydrogel Alleviated the Apoptosis and Promoted Mitophagy in the Spinal Cord Slice

**Figure 5:**
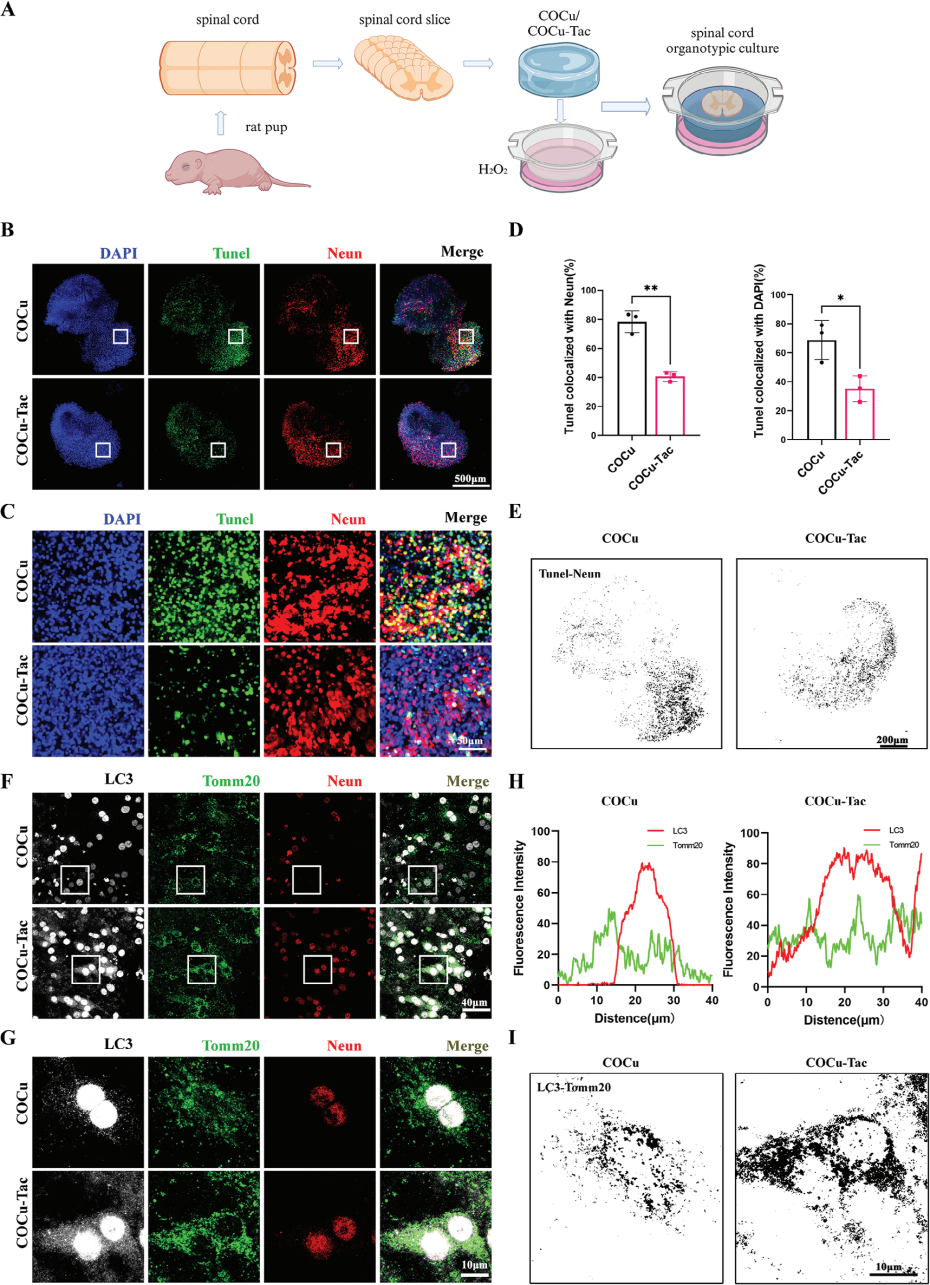
Spinal cord organotypic experiment of COCu‐Tac hydrogel. A) Schematic diagram of spinal organotypic experiment; B) Immunofluorescence of spinal cord slice. Nucleus, apoptotic cells, and neurons were labeled with DAPI, Tunel, and Neun, respectively; C) Enlarged pictures of B), Scale bar, 500 µm for original pictures and 50 µm for enlarged pictures; D) Quantification of the average level of colocalization between Tunel and Neun or Tunel and DAPI in (B) (*n* = 3), ^*^ *p* < 0.05, ^**^ *p* < 0.01, ^***^ *p* < 0.001, ns means no significance; E) Co‐localized Tunel and Neun in (B); F) Immunofluorescence of spinal cord slice. Autophagic cells, mitochondria, and neurons were labeled with LC3, Tomm20, and Neun, respectively; G) Enlarged pictures of F), Scale bar, 40 µm for original pictures and 10 µm for enlarged pictures; H) Colocalization analysis of the fluorescence intensity of LC3 and Tomm20 in (F); I) Co‐localized LC3 and Tomm20 in (G), Scale bar, 10 µm.

Serving as an ex vivo model (**Figure** **5**A), the organotypic spinal cord slice offers the flexibility and convenience of cell culture while retaining the intricate cytoarchitecture and microenvironment typical of in vivo conditions.^[^ ^35^ ^]^ Our initial investigations focused on the impact of the COCu‐Tac hydrogel on apoptosis. Co‐staining with TUNEL and NeuN highlighted neuronal apoptosis in spinal cord slices subjected to H_2_O_2_‐induced oxidative injury. The results clearly showed that COCu‐Tac hydrogel significantly reduced neuronal apoptosis. Furthermore, additional co‐staining with TUNEL and DAPI revealed that the hydrogel also curtailed overall apoptosis across the tissue (Figure 5B–E). Subsequent analysis of mitophagy within neurons demonstrated an increase in the co‐localization of Tomm20 and LC3 in the COCu‐Tac group, signaling enhanced mitophagy (Figure 5F–I). Collectively, these findings suggest that COCu‐Tac hydrogel may facilitate mitophagy in residual neurons after SCI, effectively diminishing apoptosis.

### 2.10 COCu‐Tac Hydrogel Promoted iNSCs Survival After Transplantation

**Figure 6:**
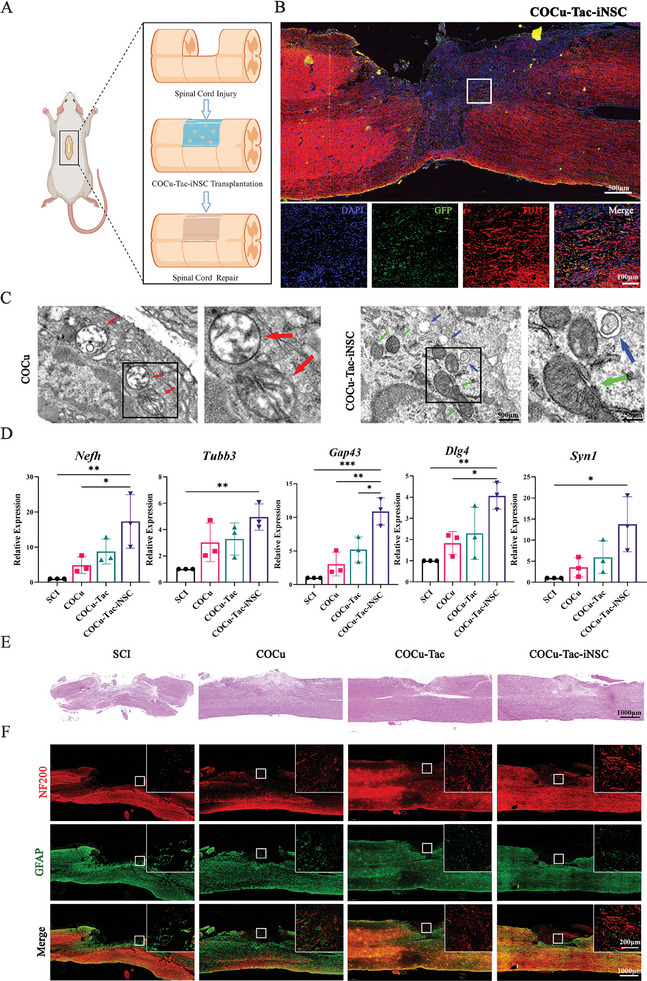
Axon Regeneration in SCI Rats after COCu‐Tac‐iNSCs Transplantation. A) Schematic representation of the COCu‐Tac‐iNSC hydrogel for SCI repair; B) The immunofluorescence staining of GFP+iNSCs in spinal cords 6 weeks after injury. iNSC and Neurons were labeled with GFP and TUJ1 respectively; C) Representative TEM images of mitochondrial morphology in SCI rats. Scale bar, 500 µm for original pictures and 200 µm for enlarged pictures; D) RT‐PCR to detect the expression levels of several axon‐related markers in the four groups 6 weeks after SCI (*n* = 3), ^*^ *p* < 0.05, ^**^ *p* < 0.01, ^***^ *p* < 0.001; E) HE Staining of the Spinal Cords in Each Group; F) The immunofluorescence staining of spinal cords 6 weeks after injury. Nerve filaments and astrocytes were labeled with NF200 and GFAP respectively.

The survival and neuronal differentiation post‐transplantation are critical for the success of iNSCs transplantation. Numerous studies suggest that NSCs predominantly differentiate into astrocytes rather than neurons.^[^ ^36^, ^37^ ^]^ To explore survival and differentiation, GFP‐labeled iNSCs were used in rats undergoing spinal cord hemisection (**Figure** **6**A; Figure S4, Supporting Information). The transplantation was performed without systemic immunosuppression. At 6 weeks post‐surgery, a significant number of viable iNSCs (GFP^+^) were observed in the COCu‐Tac‐iNSCs group, with the majority differentiating into neurons (TUJ1^+^) and a minority into astrocytes (GFAP^+^). In contrast, the COCu‐iNSCs group showed minimal iNSCs survival, predominantly differentiating into astrocytes (Figure 6B; Figure S5, Supporting Information).

### 2.11 COCu‐Tac hydrogel Suppresses the Foreign Body Reaction Following Hydrogel Transplantation

Material and cell transplantation are often accompanied by T‐cell infiltration and foreign body reaction (FBR). We assessed T cell infiltration using CD4 and CD8 markers, while GFAP and CD13 were utilized to label border‐forming astrocytes and invading non‐neural cells, including stromal and peripheral myeloid lineage cells. Additionally, we examined inflammatory infiltration and microglial activation using P2Y12R (resting microglia) and CD45 (leukocytes and reactive microglia).

The results indicated that, compared to COCu hydrogel, COCu‐Tac hydrogel significantly inhibited T cell activation following iNSC transplantation (Figure S6, Supporting Information). In the scenario of pure hydrogel implantation, the sustained release of tacrolimus effectively suppressed foreign cell invasion and reduced microglial activation, likely due to its ability to suppress T‐cell infiltration (Figure S7, Supporting Information). Research has confirmed that T cells can exacerbate inflammatory responses and activate microglia through the secretion of IFN‐γ.^[^ ^38^ ^]^ Thus, the sustained release of tacrolimus may promote the recovery of microglial homeostasis by modulating T cell activity, thereby improving the injury microenvironment and mitigating the foreign body reaction.

We further assessed the integration and degradation of the hydrogel with the host spinal cord. The results showed that the transplanted material integrates almost completely with the spinal cord by 3 weeks post‐injury and is fully integrated by 6 weeks post‐operation. In vivo degradation experiments confirmed that the implanted hydrogel degrades within 5 weeks, exhibiting a slightly slower overall trend compared to in vitro degradation. This discrepancy may be attributed to the fact that while biological responses in vivo can accelerate material degradation, surrounding tissues also infiltrate the hydrogel over time.

### 2.12 COCu‐Tac‐iNSCs Hydrogel Boosted the Regeneration of Nerve Fibers Through Mitophagy

To further investigate the role of COCu‐Tac in promoting mitophagy in vivo, we conducted a transmission electron microscope (TEM) analysis of mitochondria in the injured areas of each group. The COCu group showed numerous damaged and swollen mitochondria(red arrows), with a lack of autophagosomes. In contrast, the COCu‐Tac‐iNSCs group exhibited both morphologically normal mitochondria(green arrows) and damaged mitochondria enclosed in autophagosomes(blue arrows), indicating that the COCu‐Tac hydrogel enhanced mitophagy in transplanted iNSCs, thereby improving MQC and mitochondrial homeostasis (Figure 6C). Further analyses via RT‐PCR revealed that COCu hydrogel facilitates neural regeneration at the injury site, likely by providing a supportive ECM for neural regrowth. Additionally, the COCu‐Tac hydrogel showed improved axonal regeneration, while the COCu‐Tac‐iNSCs group demonstrated the highest expression levels of neural‐related genes and proteins, marking a significant presence of regenerating neurons (Figure 6D)

The integration of graft and host spinal tissue is crucial for reconstructing neural circuits.^[^ ^39^, ^40^, ^41^ ^]^ Post‐treatment HE staining of spinal cord tissues revealed persistent cavities in the injury site of the SCI group. In contrast, the gel‐implanted groups showed significant cavity filling, attributed to the material's effective self‐adaptation and self‐healing capabilities (Figure 6E). Immunofluorescence studies indicated that COCu‐Tac significantly promoted the span of residual neurons across the injury area after SCI. In the COCu‐Tac‐iNSCs group, the surviving iNSCs contributed to an increased content of neurons, effectively aiding the reconstruction of neural circuits within the injury area (Figure 6F).

### 2.13 COCu‐Tac‐iNSCs Hydrogel Promoted Recovery of Locomotor Function After SCI

**Figure 7:**
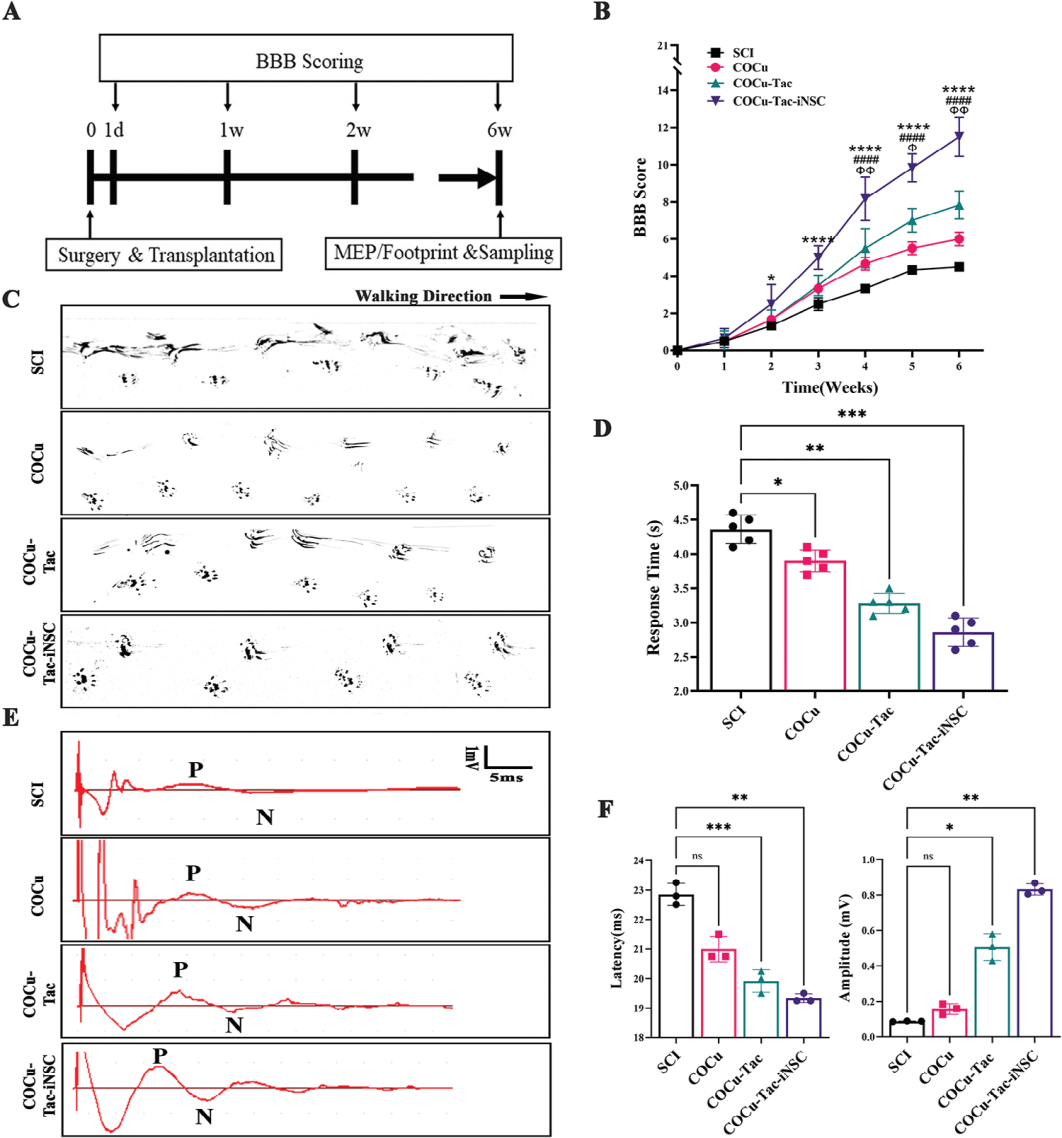
COCu‐Tac‐iNSCs Hydrogel Promotes Recovery of Locomotor Function After SCI. A) Flowchart of behavioral and electrophysiological tests; B) the Basso–Beattie–Bresnahan (BBB) score of the left hindlimb of SCI rats, ^*#ɸ^ *p* < 0.05, ^**##ɸɸ^ *p* < 0.01, ^***###ɸɸɸ^ *p* < 0.001, * means the comparison of COCu‐Tac‐iNSC group and SCI group, # means the comparison of the COCu‐Tac group and SCI group, ɸ means the comparison of the COCu group and SCI group; C) Footprints of the hindlimb in rats at 6 weeks post‐injury; D) Quantitative statistics of response time to the photothermal stimulation (*n* = 3), ^*^ *p* < 0.05, ^**^ *p* < 0.01, ^***^ *p* < 0.001; E) Evoked potentials of the sciatic nerve of the left hindlimb in rats at 6 weeks post‐injury. F) Statistics of the amplitude and latency of Evoked potentials in (E) (*n* = 3), ^*^ *p* < 0.05, ^**^ *p* < 0.01, ^***^ *p* < 0.001, ns means no significance.

To evaluate the efficacy of the COCu‐Tac‐iNSCs hydrogel in promoting functional recovery, we conducted behavioral and electrophysiological tests (**Figure** **7**A). Weekly assessments using the Basso–Beattie–Bresnahan (BBB) scale showed limited spontaneous recovery in most SCI rats at 6 weeks post‐surgery, with only two or three barely movable joints (BBB score = 5). The COCu and COCu‐Tac groups exhibited modest improvements, with BBB scores reaching seven and nine respectively, yet no permanent weight support was observed. In contrast, rats in the COCu‐Tac‐iNSCs group demonstrated significant recovery, achieving permanent weight support and, in some cases, coordinated walking (BBB score = 13) (Figure 7B).

Footprint analysis further supported these findings. By the conclusion of the experiment, untreated SCI rats still exhibited joint contractures and left indistinct tracks. Rats treated with COCu hydrogel showed recognizable footprints but relied on the dorsum of their feet for support. Meanwhile, those in the COCu‐Tac group demonstrated improved walking patterns with narrower bases and longer strides, occasionally using the soles of their feet. Most impressively, the COCu‐Tac‐iNSCs group produced complete footprints indicating restored weight‐bearing capability, with their walking base and stride showing consistent improvement, suggesting partial restoration of limb coordination (Figure 7C). Furthermore, the recovery of sensory functions was evaluated using the plantar test, which measured the response time of the hindlimb to thermalgia. Treatment with COCu‐Tac‐iNSCs hydrogel significantly reduced the response time, which revealed enhanced sensory perception associated with sensory axon regeneration (Figure 7D).

Objective assessment of neural circuit remodeling was conducted by analyzing Motor‐Evoked Potentials (MEP) 6 weeks post‐SCI. Both COCu‐Tac and COCu‐Tac‐iNSCs hydrogel treatments significantly enhanced the response by reducing latency and increasing amplitude compared to the SCI group, with the impact most pronounced in the COCu‐Tac‐iNSCs group (Figure 7E,F). The improved electrophysiological characteristics indicate that the iNSCs within the hydrogel have established polysynaptic relays capable of conducting neural signals alongside residual neurons, thus contributing to functional recovery.

### 2.14 COCu‐Tac‐iNSC Hydrogel Attenuates Gastrocnemius Muscle Atrophy After SCI

**Figure 8:**
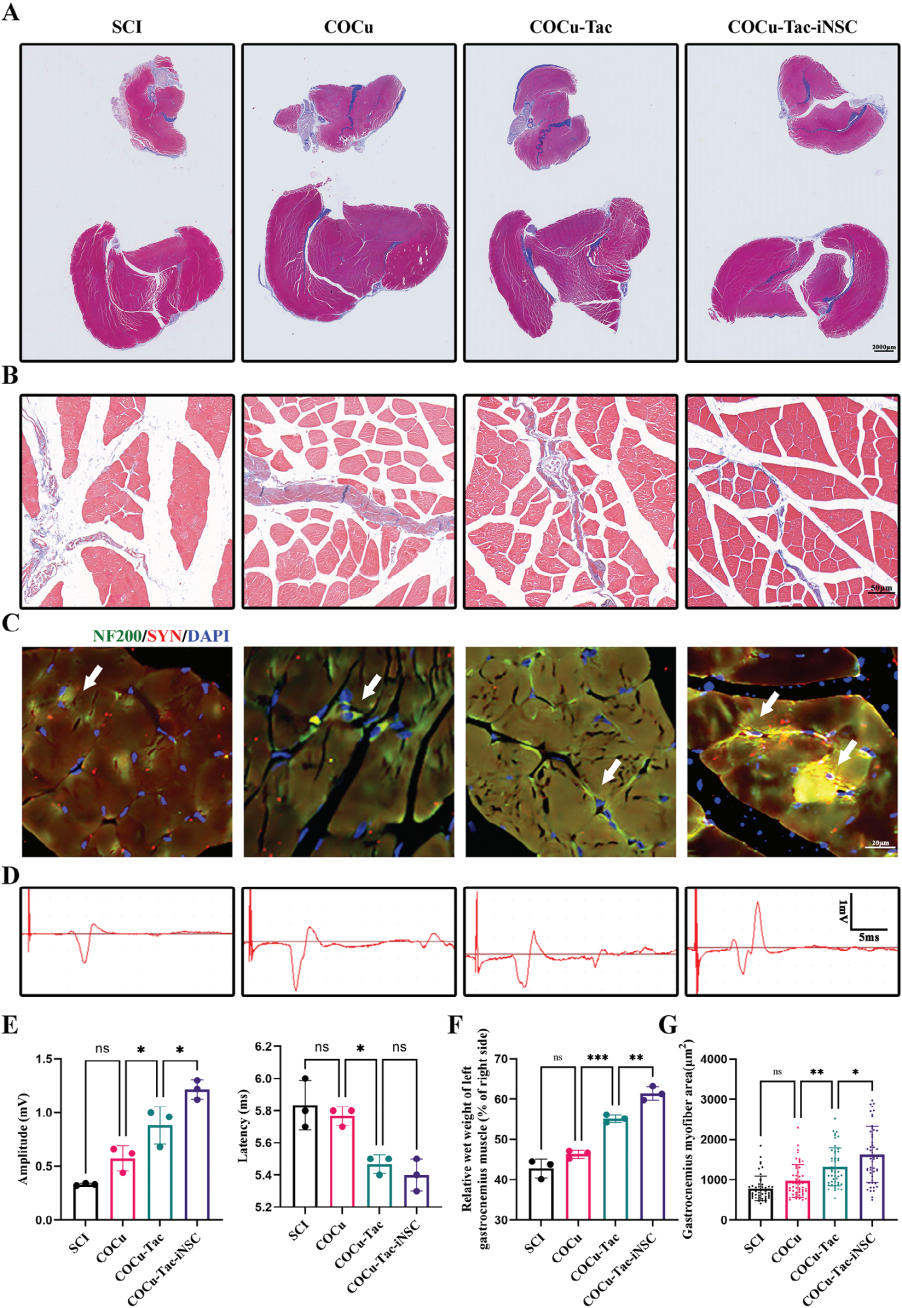
COCu‐Tac‐iNSC Hydrogel Attenuates Gastrocnemius Muscle Atrophy after SCI. A) The longitudinal view of the bilateral gastrocnemius muscles 6 weeks after injury highlighted by Masson's trichrome stain (MTS), Scale bar, 2000 µm; B) The cross‐sectional view of the left gastrocnemius muscles highlighted by MTS, Scale bar, 50 µm; C) The immunofluorescence staining of left gastrocnemius muscles. Nerve filaments and synapses were labeled with NF200 and SYN respectively, Scale bar, 20 µm; D) Evoked potentials of the gastrocnemius muscles of the left hindlimb in rats at 6 weeks post‐injury; E) Statistics of the amplitude and latency of Evoked potentials in (D) (*n* = 3); F) Quantitative statistics of the wet weight ratio of the bilateral gastrocnemius muscle (*n* = 3), (G) Quantitative statistics of the gastrocnemius myofiber area, (*n* = 50) ^*^ *p* < 0.05, ^**^ *p* < 0.01, ^***^ *p* < 0.001, ns means no significance.

Denervation‐induced atrophy of the gastrocnemius muscle following SCI significantly contributes to motor function loss and serves as a crucial indicator of functional recovery.^[^ ^42^ ^]^ Histological examination, employing Masson's trichrome stain, allowed for gross observation of the gastrocnemius muscles. Notably, the muscle on the injured side exhibited significant atrophy compared to the contralateral side, particularly within the SCI group (**Figure** **8**A). Additionally, substantial collagen deposits were observed bilaterally in the SCI group. In contrast, the COCu‐Tac‐iNSCs treatment group showed markedly reduced signs of atrophy and collagen deposition, attributable to enhanced nerve reinnervation. Larger muscle fibers and fewer collagen deposits were observed compared to other groups. Cross‐sectional analysis of the left gastrocnemius muscle demonstrated a higher muscle fiber density, further confirming that COCu‐Tac‐iNSCs implantation facilitated muscle fiber recovery (Figure 8B).

To assess muscle innervation, we employed NF200 and synaptophysin markers on the gastrocnemius muscle. Post‐COCu‐Tac treatment, levels of NF200 and synaptophysin significantly increased. Specifically, in the COCu‐Tac‐iNSCs group, the axonal termini of the nerves innervating the muscle displayed markedly increased density (Figure 8C). Electrophysiological assessments of the gastrocnemius muscle further confirmed that COCu‐Tac‐iNSCs improved neural innervation of the hind limb muscles (Figure 8D,E). Moreover, increased muscle mass and gastrocnemius myofiber area further validated the role of COCu‐Tac‐iNSCs in promoting muscle fiber regeneration post‐SCI (Figure 8F,G).

Additionally, visceral HE staining and hemolysis experiments confirmed the safety of the material (Figure S9, Supporting Information). Overall, these results substantiate the material's safety and efficacy in promoting neural regeneration.

## 3 Discussion

Successful delivery of transplanted cells and maintenance of cell viability after SCI are critical challenges faced by many cell‐based therapies. Previous studies have explored various tissue engineering approaches for cell transplantation. However, post‐transplantation, scaffold materials are prone to rupture due to various mechanical stresses, which can hinder effective neural regeneration.^[^ ^26^, ^43^, ^44^ ^]^ Additionally, rejection following cell transplantation requires the systemic use of immunosuppressive agents, which can lead to severe adverse reactions.^[^ ^10^, ^45^, ^46^ ^]^ Moreover, energy metabolism disruption after SCI restricts the survival of transplanted cells, further limiting the feasibility of such approaches.^[^ ^7^ ^]^ In response, we have designed a hydrogel scaffold with self‐healing properties and controlled release of the tacrolimus. We have demonstrated that this scaffold can target FKBP52 to induce mitophagy in transplanted iNSCs. Therefore, our strategy improves cell survival by addressing three key underlying causes of cell loss: i) anoikis due to loss of ECM and scaffold rupture, ii) graft rejection, and iii) energy metabolism disturbances caused by mitochondrial dysfunction.

Tacrolimus, a commonly used clinical immunosuppressant, has been commercialized in over 70 countries. However, its systemic administration is associated with several side effects. Long‐term use of tacrolimus can lead to complications such as infections, kidney diseases, neurotoxicity, diabetes mellitus, and alterations in gut microbiota, with adverse effects occurring even at low dosages.^[^ ^29^, ^47^, ^48^ ^]^ Additionally, local vascular damage post‐SCI, coupled with inadequate blood supply, often results in suboptimal drug concentrations at the injury site, necessitating higher overall dosages.^[^ ^49^ ^]^ Consequently, the development of locally controlled release systems using advanced biomaterial technologies has emerged as a new research direction. Previous research has employed localized tacrolimus release to improve transplantation outcomes. Wu et al. designed a peptide‐based hydrogel with immune‐responsive properties for the sustained release of tacrolimus. This hydrogel releases tacrolimus through degradation by protein tyrosine kinases activated by T cells, effectively prolonging liver transplant survival times.^[^ ^29^ ^]^ Recently, two controlled‐release systems for tacrolimus have been introduced to improve graft survival in allogeneic spinal transplantation. Zhao et al. engineered a sustained tacrolimus‐release collagen hydrogel (Col/Tac), achieving prolonged tacrolimus release, with 15% remaining after 26 days in vitro.^[^ ^49^ ^]^ However, the clinical applicability of allogeneic adult spinal cord tissue transplantation remains challenging due to ethical and donor source issues. Sevc et al. developed a subcutaneous pellet for the controlled release of tacrolimus, which enhances the survival of human neural precursors post‐spinal cord injury (SCI). This system provides long‐term release of tacrolimus for up to 3 months in vivo.^[^ ^50^ ^]^ Nonetheless, it still functions as a systemic medication and does not avoid the associated side effects.

Prolonging the sustained release of tacrolimus can enhance transplantation success rates. For SCI applications, however, releasing free drugs from a hydrogel matrix may not always provide sufficient temporal control for sustained drug release. The inverse relationship between hydrogel pore size and stiffness means that the small pore sizes required for sustained release may result in gels that are too stiff to be safely implanted on or within the soft and fragile spinal cord tissue.^[^ ^51^ ^]^ Therefore, in future experiments, we could design a tacrolimus release switch for COCu hydrogel or attach tacrolimus to hydrogels via degradable covalent bonds. Alternatively, loading tacrolimus into sustained‐release microspheres could achieve prolonged tacrolimus release.

MQC is critical for the survival and functionality of transplanted cells and organs. Mitochondrial dysfunction can lead to senescence in mesenchymal stem cells post‐transplantation, thereby reducing their therapeutic potential.^[^ ^52^ ^]^ Neurons have even higher demands for MQC due to their diverse functions, including maintaining membrane potential, neurotransmitter release, circulation, and axoplasmic transport, which require substantial energy.^[^ ^53^, ^54^ ^]^ The regeneration of axons is particularly reliant on mitochondrial function, as it necessitates ≈50% of the cell's ATP.^[^ ^55^ ^]^ To date, no studies have explored the role of tacrolimus in improving graft mitochondrial homeostasis. Our study revealed that COCu‐Tac enhances mitophagy in iNSCs, thereby improving cellular MQC. This intrinsic mechanism is associated with the regulatory effect of FKBP52 on AKT.

Our study revealed that knocking down FKBP52 leads to reduced AKT phosphorylation and enhanced mitophagy, consistent with the effects of tacrolimus treatment. Conversely, inhibiting the non‐PPIase function of FKBP51 using SaFit1 resulted in increased AKT phosphorylation and diminished mitophagy. This suggests that FK506 may inhibit AKT phosphorylation and promote mitophagy through differential effects on FKBP51 and FKBP52. This differential regulation is also observed in the modulation of microtubule stability by FK506. FKBP52 antagonizes Tau (Tubulin‐associated unit) ‐promoted tubulin assembly, while FKBP51 promotes microtubule stabilization via Tau and Hsp90.^[^ ^56^ ^]^ However, neurons treated with FK506 exhibited only microtubule‐promoting effects. In undifferentiated neurons, FKBP52, as part of the FKBP52·Hsp90·p23 complex, is concentrated in a perinuclear ring. Upon FK506 treatment, this complex disassembles, and FKBP52 relocates to the growth cones, where it promotes microtubule formation. Meanwhile, FKBP51 remains in the cell body, forming a new perinuclear ring with Hsp70, replacing FKBP52.^[^ ^57^ ^]^ This alteration in the positioning of FKBP51/52 following FK506 treatment may contribute to their distinct responses in modulating AKT phosphorylation.

However, there are some limitations to our current study.

First, the duration of the study was limited to 6 weeks post‐spinal cord injury, while extracellular matrix (ECM) remodeling and neural circuit reconstruction can persist for a year or longer.^[^ ^24^ ^]^ Moreover, while our study minimizes systemic exposure through localized tacrolimus release, potential neurotoxicity remains a concern, as tacrolimus can lead to headaches, tremors, seizures, and sensory disturbances, even at therapeutic concentrations.^[^ ^11^ ^]^ Therefore, it is necessary to explore the long‐term effects of tacrolimus on local tissue homeostasis over an extended period. Second, although this study preliminarily explored the foreign body response post‐material transplantation, it did not further specify the types of CD13‐positive cells post‐transplantation. Research has shown that P2Y12R‐positive microglia contribute to spinal cord injury repair.^[^ ^58^ ^]^ Thus, CoCu‐Tac may promote neural regeneration by restoring microglial homeostasis. Further studies are needed to explore the specific roles of infiltrating stromal and immune cells post‐material transplantation, as well as the regulatory effects of microglial activation. Lastly, despite extensive research on iNSC transplantation following SCI, clinical translation is impeded by risks associated with tumorigenicity and potential genomic instability. To address these concerns, studies suggest that γ‐secretase inhibitor 5 (GSI) and the herpes simplex virus type 1 thymidine kinase (HSVtk) gene may mitigate the tumorigenic potential of iNSCs.^[^ ^59^ ^]^ Given these challenges, further safety assessments and large‐scale animal studies are essential to evaluate the feasibility of clinical applications.

Our study introduces an innovative COCu‐Tac‐iNSCs transplantation system and unveils a novel mechanism wherein tacrolimus targets FKBP52 to promote mitophagy, thus safeguarding the graft. However, the precise pathways through which tacrolimus targets FKBP52 to regulate AKT phosphorylation and the direct antagonistic effects of FKBP51/52 remain insufficiently explored. Additionally, other research has shown that FKBP51 can activate mitophagy by suppressing PPAR‐γ after multiple sclerosis.^[^ ^60^ ^]^ Furthermore, FKBP38 recruits LC3A to mediate Parkin‐independent mitophagy^[^ ^61^ ^]^ and prevents apoptosis during the mitophagy process.^[^ ^62^, ^63^ ^]^ Hence, the regulatory effects of tacrolimus on other FKBPs warrant further investigation. This finding underscores the importance of mitochondrial health in neural repair and positions tacrolimus as a dual‐function agent that not only prevents graft rejection but also enhances cellular viability through the promotion of mitophagy. Moreover, the modular nature of this hydrogel system facilitates the customization of transplantation scaffolds for various cell types and could be adapted for other clinical conditions beyond SCI.

## 4 Experimental Section

### Materials of the COCu Hydrogel

CMCS was purchased from Shanghai Dibai Chemical Technology Co., Ltd. Chitosan oligosaccharide (COS, Mw 800–1000 Da), and chitosan (Mw 50 000–60 000 Da) were provided by Dalian Meilun Biotechnology Co., Ltd. 4‐FA, 4‐dimethylamino pyridine (DMAP), dimethyl sulfoxide (DMSO), tetramethylene pentaamine (TEPA) and sodium borohydride (NaBH_4_) were purchased from Shanghai Aladdin Biochemical Technology Co., Ltd. Dicyclohexyl carbon diimide (DCC) was purchased from Shanghai Bide Medical Technology Co., Ltd. Citric acid, acetone, copper chloride dihydrate (CuCl_2_ H_2_O) was purchased from Tianjin Damao Chemical Reagent Factory.

### Preparation of OCS

OCS was synthesized following a previously established method in the laboratory.^[^ ^12^ ^]^ Initially, 2.5 g of chitosan oligosaccharide was dissolved in 50 mL of DMSO, followed by the addition of 4.5 g of p‐formylbenzoic acid dissolved in 20 mL of DMSO to the stirring solution. Subsequently, 0.1 g of 4‐dimethylaminopyridine (DMAP) and 1.68 g of DCC were added. The entire mixture was allowed to react at room temperature for 24 h. The resulting product was obtained by precipitation, washing, and filtration using deionized water for three repetitions, followed by freeze‐drying for storage at 4 °C.

### Preparation of Carbon Dots Copper (CDs‐Cu)

CDs‐Cu was synthesized using a previously established method in the laboratory.^[^ ^64^ ^]^ Briefly, 0.04 g of citric acid was dissolved in 40 mL of water, and 0.16 mL of TEPA was added. The mixture was stirred and heated to 180 °C for 6 h, followed by cooling to room temperature to obtain a carbon quantum dot solution (CDs).

A blue‐purple mixed solution was prepared by combining 40 mL of 0.1 mol/L CuCl2·2H2O solution with an equal volume of the CDs solution. To this mixture, 80 g of NaOH was slowly added under stirring, creating the blue‐purple solution. The mixture was transferred to a round‐bottom flask and heated to 70 °C in an oil bath. Subsequently, 1.3 g of reducing agent NaBH4 was added, and the reaction was carried out for 3 h. After the appearance of black suspended particles, the product was subjected to centrifugation, washing, and filtration for four repetitions, followed by drying at 60 °C to obtain CDs‐Cu.

### Preparation of the COCu Hydrogel

Hydrogels were prepared based on our previous work.^[^ ^12^ ^]^ In 10 ml of deionized water, 0.06 g of aldehyde‐modified chitosan oligosaccharide and 0.6 g of carboxymethyl chitosan were added and stirred. A certain amount of tacrolimus was then introduced. Subsequently, 4 mg of mesocrystal copper powder was added, and the mixture was stirred at room temperature for 10 h to obtain the blue copper‐based drug‐loaded hydrogel. Additionally, a blank copper‐based hydrogel was prepared using the same method without adding tacrolimus.

Hydrogels were prepared based on our previous work.^[^ ^12^ ^]^ In 10 mL of deionized water, 0.06 g of aldehyde‐modified chitosan oligosaccharide and 0.6 g of carboxymethyl chitosan were added and stirred. Tacrolimus (82.2 mg) was dissolved in 100 mL of ethanol to prepare the tacrolimus solution, from which 100, 200, 300, and 400 µL were added to the above solution, respectively. After adding 4 mg of mesoporous copper powder, the solution was stirred at room temperature for 10 h, yielding blue copper‐based drug‐loaded hydrogels with FK506 concentrations of 10, 20, 30, and 40 µm. Additionally, a blank copper‐based hydrogel was prepared using the same method without the addition of tacrolimus.

### Material Characterizations

The infrared spectra of COS, 4‐FA, and OCS were measured using a Nicolet 6700 FTIR spectrometer from Thermo Fisher Scientific. The 1H NMR spectra of chitosan oligosaccharide and aldehyde‐modified chitosan oligosaccharide were recorded using a Bruker Biospin AG AV600 NMR spectrometer with deuterated DMSO as the solvent. XRD analysis of carbon dots copper, CMCS, and copper‐based hydrogel was performed using a Rigaku D/max‐IIIA X‐ray diffractometer (Cu Kα (1.542 Å) radiation) from Rigaku Corporation, with a fixed 2θ range of 10–80. The microstructure of copper hydrogel was observed using an SEM XL‐30‐ESEM from FEI Company, and samples were gold‐coated before testing.

### Rheology Test

Hydrogels were molded into cylindrical sheets (diameter: 25 mm, thickness: 1.2 mm) using specific molds. The storage modulus (G’) and loss modulus (G″) of hydrogels were measured at 37 °C using an Anton Paar MCR502 rheometer. Frequency sweep tests were conducted at a constant strain of 1%, with angular frequencies ranging from 0.01 to 100 rad s^−1^. Strain sweep tests were conducted at a constant frequency of 1 rad s^−1^, with strains ranging from 0.01% to 100%. The viscosity of hydrogels was measured at 37 °C and 1% strain, with shear rates ranging from 0.1 to 100 s^−1^.

### Self‐Healing and Self‐Adapting Performances of Hydrogels

To assess the self‐healing and self‐adapting properties of the hydrogels, a 4 cm‐long COCu hydrogel was cut in half, with one half dyed using methyl orange. These halves were then carefully aligned along the cut edge at room temperature, requiring no external assistance. To assess the self‐adapting properties, a small ball‐filling experiment was performed. The hydrogel was placed over a layer of blue glass beads, each with a diameter of 14 mm, and the movement and filling behavior of the gel was observed.

### In Vitro Gel Swelling and Degradation

The swelling ratio of hydrogels was measured using the immersion method. Freeze‐dried hydrogel (0.035 g) was immersed in pH 7.4 PBS at 37 °C, and the surface water was weighed at specific time points until the gel weight no longer changed. The swelling ratio (Es) was calculated using the formula: Es = (Ws/Wd), where Es was the swelling ratio, Ws was the swelling weight of the hydrogels, and Wd was the initial dry weight of the hydrogels.

Degradation experiments involved immersing freeze‐dried hydrogels in pH 7.4 PBS at 37 °C. The dry weight of the gels was measured at specific time points. The degradation rate (ER) was calculated using the formula: ER = (Wi/Wf), where ER was the degradation rate, Wi was the initial dry weight of the gel, and Wf was the dry weight of the remaining gel after degradation.

### In Vitro Release of Tacrolimus from the COCu‐Tac Hydrogels

A specific amount of COCu‐Tac hydrogel was placed in a dialysis bag and immersed in a 50 mL PBS solution containing 0.1% Tween 80 at pH 7.4. The system was placed in a shaking incubator at 37 °C and 110 rpm. At specific time points, 2 mL of the release buffer was collected for analysis, and an equivalent volume of fresh PBS solution was added. The drug concentration was analyzed using high‐performance liquid chromatography (HPLC).

### iNSCs Generation and Culture

Human iPSCs (hiPSCs) were identified as previously reported.^[^ ^65^, ^66^ ^]^ Culture plates were pre‐coated with Matrigel (Corning) and hiPSCs were propagated as colonies in mTeSR medium (Stem Cell Technologies, USA) at 37 °C in a 5% CO_2_ incubator. The protocol applied to generate sustainable spinal cord iNSCs was based on the method proposed before.^[^ ^4^ ^]^ In brief, when cells were at ≈70% confluence, the mTeSR medium was replaced with the neural induction medium (NIM). The medium was refreshed every day, and cells were split 1:3 with Accutase (Gibco, USA). 10 d after neural induction, NIM was replaced with the neural maintenance medium (NMM), and the plate coating was changed from Matrigel to poly‐l‐ornithine (Sigma)/laminin (Sigma, USA) ‐coated plates.

For spontaneous differentiation, iNSCs were seeded on Poly‐D‐lysine (Gibco, USA) coated plates as neural spheres in the differentiation medium. Immunocytochemistry was performed 7 d after differentiation. 200 µm H_2_O_2_ was used to simulate oxidative stress during SCI.

### Measurement of Cell Viability and Biocompatibility

The cell viability of the iNSCs was detected using a live/dead cytotoxicity kit (calcein‐AM/PI, Invitrogen, USA) and cell counting kit‐8 solution (CCK‐8, Dojindo, Japan). For live/dead analysis, iNSCs in different groups were washed with PBS and double‐stained with calcein‐AM and propidium iodide for 20 min at room temperature. Samples were visualized using a laser confocal scanning microscopy (Leica, Germany). CCK8 solution (1:10) was added to each group and the optical density (OD) was calculated using an enzyme‐labeling instrument (Multiskan FC, Thermo) at a wavelength of 450 nm. The cell viability was calculated using the relative percentage compared to the control groups. Biocompatibility was further verified with the hemolytic test. Fresh blood samples were co‐incubated with CMC hydrogel for 4 h at room temperature. PBS and Triton‐100X were used as blank and positive groups. All samples were centrifugated at 12 000 g for 5 min at 4 °C and the absorbance was measured for analysis. Hemolysis (%) = (Sample absorbance–Negative control)/(Positive absorbance–Negative control) × 100%

### Measurement of Intracellular ROS and Mitochondrial ROS

The intracellular ROS levels were evaluated using a Total Reactive Oxygen Species Assay Kit (Beyotime Biotechnology, China). The iNSCs in different groups were pre‐labeled with a probe for 15 min at room temperature according to the manufacturer's protocol. Mitochondrial ROS levels were detected using the fluorescent dye MitoSOX Green (Invitrogen, USA). Cells were incubated in the medium with MitoSOX Red for 30 min according to the manufacturer's protocol. Flow cytometry analysis was performed using the FACS instrument (BD, USA)

### Measurement of Mitochondrial Membrane Potential (MMP)

MMP was measured using a mitochondrial membrane potential assay kit with JC‐1 (Beyotime Biotechnology, China). In brief, cells were incubated with JC‐1 staining solution for 30 min and washed with JC‐1 staining buffer. Flow cytometry analysis was performed using the FACS instrument (BD, USA). Normal mitochondria with JC‐1 aggregates produced red fluorescence, and depolarized mitochondria with JC‐1 monomers produced green fluorescence.

### Measurement of Intracellular ATP

Intracellular ATP content was measured using an Enhanced ATP Assay Kit (Beyotime Biotechnology, China) as the manufacturer's instructions. Briefly, cellular supernatant was added to a 96‐well plate, quickly mixed with an equal volume of reagent for 2 s, and luminescence was recorded using an enzyme‐labeling instrument (Multiskan FC, Thermo)

### Mitophagy Assay

The mitophagy was determined by the mitophagy detection kit (Dojindo, Japan) and mitochondria were tracked using Mitotracker(Dojindo, Japan) as the manufacturer's instructions. In brief, cells were incubated with Mitophagy Dye for 30 min before H_2_O_2_ injury. After H_2_O_2_ injury, iNSCs were incubated with Mitotracker AND Lysosome Dye for 20min. All samples were visualized using a laser confocal scanning microscopy (Leica, Germany).

### Immunofluorescence Staining

Cells were fixed in 4% paraformaldehyde for 30 min at room temperature and then incubated in a blocking buffer (3% bovine serum albumin (BSA) and 0.1% Triton X 100 in PBS) for 1 h at room temperature. The samples were incubated with corresponding primary antibodies at 4 °C overnight, followed by the secondary antibody at room temperature for 1 h. Finally, DAPI (Beyotime, China) was used to stain the cell nuclei. Cells were washed three times for at least 5 min with fresh PBS between each step. All samples were visualized using a laser confocal scanning microscopy (Leica, Germany). The dilution ratios of primary antibodies were listed below, Rabbit anti‐TUJ1(1:1000;Abcam, ab18207), chicken anti‐GFAP(1:5000;Abcam, ab4674), Rabbit anti‐MBP(1:5000;Abcam, ab218011), rabbit anti‐LC3(1:1000;CST, #12741), rat anti‐Neun (1:1000;Abcam, ab279297), mouse anti‐Tomm20 (1:1000;Abcam, ab56783), chicken anti‐GFP(1:3000;Abcam, ab13970), mouse anti‐NF200 (1:500;sigma, N5389), Rabbit anti‐Synaptophysin (1:1000;Abcam, ab32127), Rabbit anti‐Nanog (1:1000;Abcam, ab109250), mouse anti‐OCT4(1:1000;Abcam, ab184665), Rabbit anti‐CD4(1:1000;Abcam, ab133616), mouse anti‐CD8(1:1000;Abcam, ab33786)

### Real‐Time PCR

Total RNA was extracted using an RNA isolation and purification Kit (Omega, China) and reverse transcribed into cDNA by PrimeScript reverse transcriptase (Takara, Japan) according to the manufacturer's protocol. RT‐PCR was performed using LC‐480 and gene expression was calculated using the 2^−ΔΔCt^ method, with *ACTIN* used as an internal control. The primers used in RT‐PCR are listed in Table S1, (Supporting Information).

### Western‐Blot

The cells/tissue for testing were washed with PBS and lysed with RIPA lysis buffer (Beyotime, China) containing protease inhibitor cocktail and phosphatase inhibitor cocktail (Beyotime, China). After incubation on ice for 30 min, the lysates were centrifuged at 21 000 rpm for 15 min at 4 °C, and the supernatant was collected. Total protein concentration was determined using the BCA assay kit (Thermo, USA). The supernatants were denatured by heating with 5x loading buffer (Beyotime, China) at 100 °C for 5 min, separated by SDS‐PAGE electrophoresis, and transferred to polyvinylidene fluoride membranes (Pierce). The membranes were blocked with 5% nonfat milk in PBST for 1 h at room temperature, then incubated with primary antibody overnight at 4 °C. Secondary antibodies were incubated for 1 h at room temperature the next day, and blots were revealed using ECL reagent (Thermo Fisher Scientific, USA). GAPDH was used as an internal control. The dilution ratios of primary antibodies were listed below, and the GAPDH (1:4000; Abcam, ab8245, 37KD) was included inside as the loading control, rabbit anti‐AKT(pan)(1:1000;CST, #5373, 60KD), rabbit anti‐p‐AKT (Ser473) (1:1000;CST, #5012, 60KD), rabbit anti‐p‐AKT (Thr308) (1:1000;CST, #5106, 60KD), rabbit anti‐Pink1(1:1000;CST, #85325, 50KD), mouse anti‐Parkin(1:1000;CST, #4211, 50KD), rabbit anti‐LC3(1:1000;CST, #12741, 14 KD, 16KD).

### Co‐Immunoprecipitation

Cells were lysed in lysis buffer supplemented with protease and phosphatase inhibitors. Lysates were clarified by centrifugation at 14 000 g for 15 min at 4 °C. Pre‐cleared lysates were incubated with 1–5 µg of primary antibody overnight at 4 °C, followed by a 2‐h incubation with 30 µL of protein A/G agarose beads. Beads were washed with lysis buffer and eluted with 2x SDS‐PAGE sample buffer. Eluates were analyzed by SDS‐PAGE and immunoblotting.

### Ex Vivo Study

The protocol applied was a modification of the interface method described by Chen et al.^[^ ^35^ ^]^ Rat fetal spinal cord tissues (3–5 d postnatal) were collected under a microscope. Tissues were then cut into 200 µm cross sections with a McIlwain chopper (Ted Pella, USA) for ex vivo spinal slice culture (organotypic slice culture).

The spinal slices were placed on the 0.4 µm semi‐porous membrane (Millipore) pre‐loaded with CMC hydrogel and the lower chamber was filled with culture medium (50% mem, 25% horse serum, 25%HBSS, 6.5 mg Ml−^1^ D‐glucose). 200 µm H_2_O_2_ was used to simulate oxidative stress during SCI. Tunel assay and immunohistochemistry were performed after the 3d organotypic slice culture.

### Animals and Surgical Procedures

Animals' preparation and surgery procedures were based on methods as described before.^[^ ^67^ ^]^ Adult female Sprague–Dawley rats (provided by the Guangdong Medical Laboratory Animal Center) weighing 200–220 g were employed and maintained for 7 d before any experiment to adjust to the laboratory environment. After anesthetizing with 2.5–3% isoflurane, laminectomy was performed on all rats to expose the spinal cord at T9. Then a left‐lateral hemisection lesion was performed on the injury groups. Briefly, following T9 dorsal laminectomy, the dura was removed and a 2‐mm‐long block of the left spinal cord was excised using iridectomy scissors under a microscope. Hydrogel was transplanted in the lesion area. The muscles and skin were then sutured immediately. Rats whose BBB score did not drop to 0 after surgery were excluded. Bladders of animals were manually voided three times per day for 2 weeks and penicillin (50000 unit kg^−1^ day^−1^, Jusheng, PRC) was injected subcutaneously for 3 d. All rats underwent weekly behavioral analysis and were observed for 6 weeks post‐grafting.

For in vivo degradation assessment of the hydrogel, COCu‐Tac hydrogels were implanted subcutaneously. Briefly, the animals were anesthetized, and a 1 cm incision was made on the back. A pocket was created using scissors, and four separate gels (0.5 g each) were implanted subcutaneously before closing the wound. At designated time points, the rats were sacrificed, and the remaining gels were rinsed in HBSS, dehydrated, and weighed.

### Assessment of Motor Function Recovery

The recovery of motor function was assessed by Basso, Beattie, and Bresnahan (BBB) scores and walking tracks analysis. BBB scores were assessed weekly by two trained observers post‐operation. The motor function of the hindlimbs was scored according to the standards proposed by Basso et al. ranging from 0 (no ankle movement) to 21 (complete functional recovery).^[^ ^68^ ^]^ Walking tracks analysis was performed in the final week. Briefly, all rats were trained to walk across a narrow walkway (20 cm wide by 80 cm long white paper) with both hind paws covered with black ink (non‐toxic) to record the footprints. The width of the area between the left and right hind paws was determined as the base of support. Stride length was characterized as the distance between the center pads of two adjacent footprints on the same side. The outcomes were determined by the average of three sequential steps.

### Electrophysiological Assessment

Motor evoked potentials (MEP) were detected 6 weeks after injury to examine the motor nervous system recovery of each group using the BL‐420 Data Acquisition Analysis System (TECHMAN SOFT, China). The rats were anesthetized with 2.5–3% isoflurane, and the lesion area was exposed. To assess spinal cord neurocircuitry reconstruction, stimulation electrodes were inserted into the left‐lateral spinal cord, 5 mm rostral to the lesion area. Recording electrodes were inserted into the sciatic nerve of the left hindlimbs. To assess gastrocnemius muscle atrophy, stimulation electrodes were inserted into the left sciatic nerve, and recording electrodes were inserted into the left gastrocnemius muscle. The ground electrode was placed in the fur of the tail. MEPs were evoked by electrical stimulation delivered through single rectangle pulses (duration: 5 ms; frequency: 1 Hz; voltage density: 5 V for the spinal cord, 8 V for the sciatic nerve). Amplitudes and latencies were obtained to assess nerve conduction function.

### Histological and Immunofluorescence Analysis

After the electrophysiological assessment, rats were sacrificed and the spinal cords and the gastrocnemius muscle were harvested. The rats were deeply anesthetized and perfused with iced PBS, followed by 4% paraformaldehyde. The spinal cords containing the entire injury area were dissected carefully and fixed at 4 °C in paraformaldehyde for at least 24 h. The 7 mm‐thick longitudinal and cross sections of the spinal cord were obtained by a cryostat microtome and were kept at −80 °C before use. For immunofluorescence, the sections were washed with PBS three times and permeabilized and blocked with PBS containing 0.5% Triton X‐100 and 5% BSA. The following procedures were performed as previously described.

### Statistics

Statistical analyses were performed by the statistical software SPSS 20.0. Data obtained in different experiments were presented as means ± standard deviations (SDs). Group comparisons were tested via unpaired *t*‐tests (Mann–Whitney) or one‐way analysis of variance (ANOVA), and a *p*‐value < 0.05 was considered statistically significant.

## Conflict of Interest

The authors declare no conflict of interest.

## Author Contributions

Z.M.T., H.‐J.H., and C.C.C. contributed equally to this work. B.L., G.B.J., and L.M.R. conceptualized the study. Z.M.T., H.J.H., C.C.C., C.C.Y., and H.L. developed the methodology. Z.M.T., H.J.H., T.H., C.C.C., C.C.Y., and H.L. conducted the investigation. Z.M.T., H.J.H., and T.H. created the visualization. L.B., G.B.J., and L.M.R. supervised the project. Z.M.T. and H.J.H. wrote the original draft. Z.M.T., H.J.H., B.L., G.B.J., and L.M.R. reviewed and edited the manuscript.

## Ethics Statement

All cell research protocols for this study were approved by the Ethics Committee of Sun Yat‐sen University. Informed consent was obtained from healthy volunteers prior to obtaining specimens. All samples were treated as stated in the approved ethical application and the research methods were carried out adhering to the relevant guidelines. All animal experimental procedures were approved by the Animal Ethics Committee of South China Agricultural University (2021d043).

## Supporting information



Supporting Information

## Data Availability

The data that support the findings of this study are available from the corresponding author upon reasonable request.
