# Supplementary material for: Self‐Healing COCu‐Tac Hydrogel Enhances iNSCs Transplantation for Spinal Cord Injury by Promoting Mitophagy via the FKBP52/AKT Pathway
Source: Adv Sci (Weinh). 2024 Nov 25;12(3):2407757. doi: 10.1002/advs.202407757 (PMC11744648; doi:10.1002/advs.202407757)
Supplement: Supplementary file 1 — Supporting Information [file ADVS-12-2407757-s001.docx]

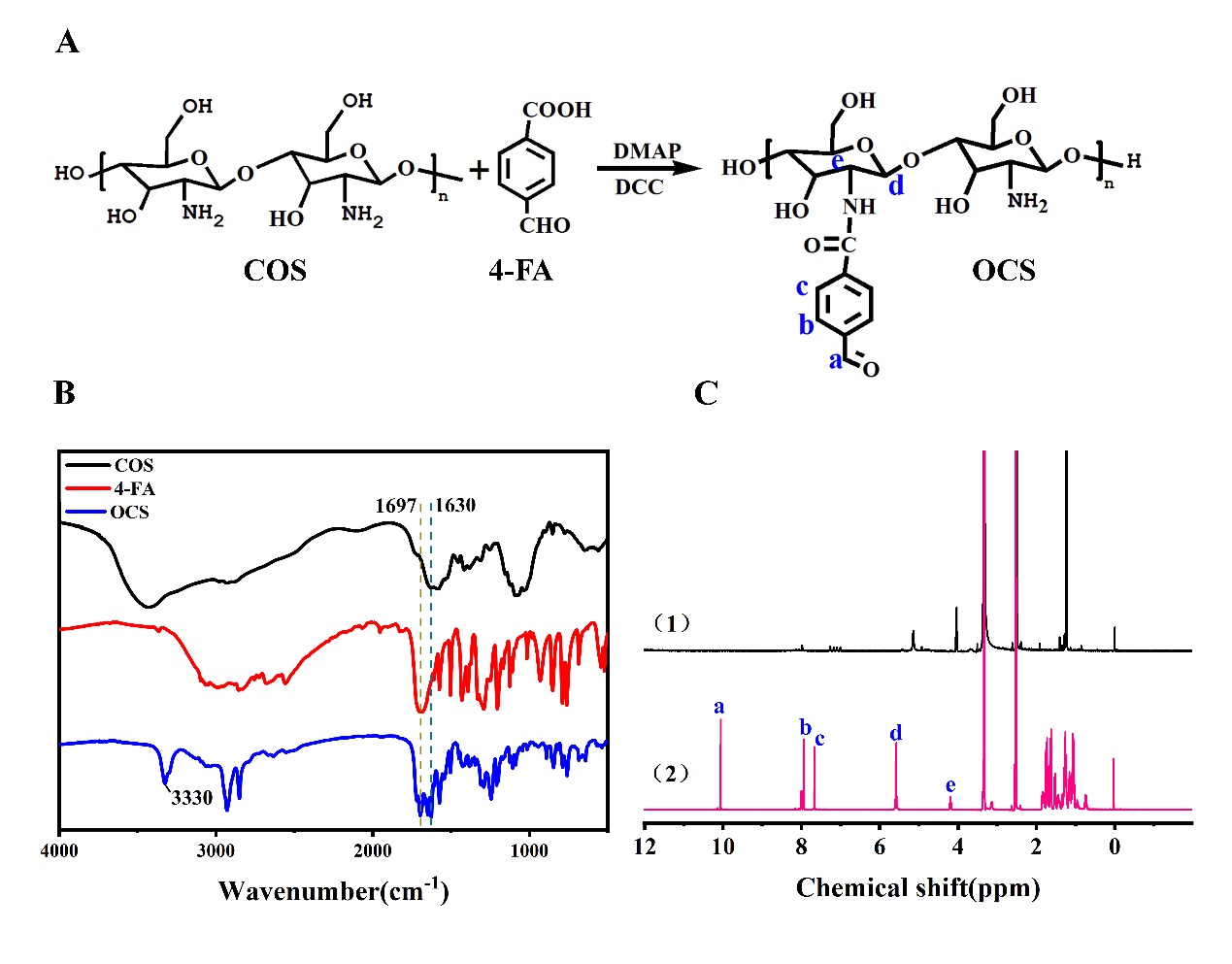


**Fig. S1 The Synthesis of OCS-CHO.**

(A) Chemical equation for the synthesis of OCS-CHO; (B) Infrared spectra of OCS, 4FA, and OCS-CHO; (C) Hydrogen NMR spectra of OCS and OCS-CHO


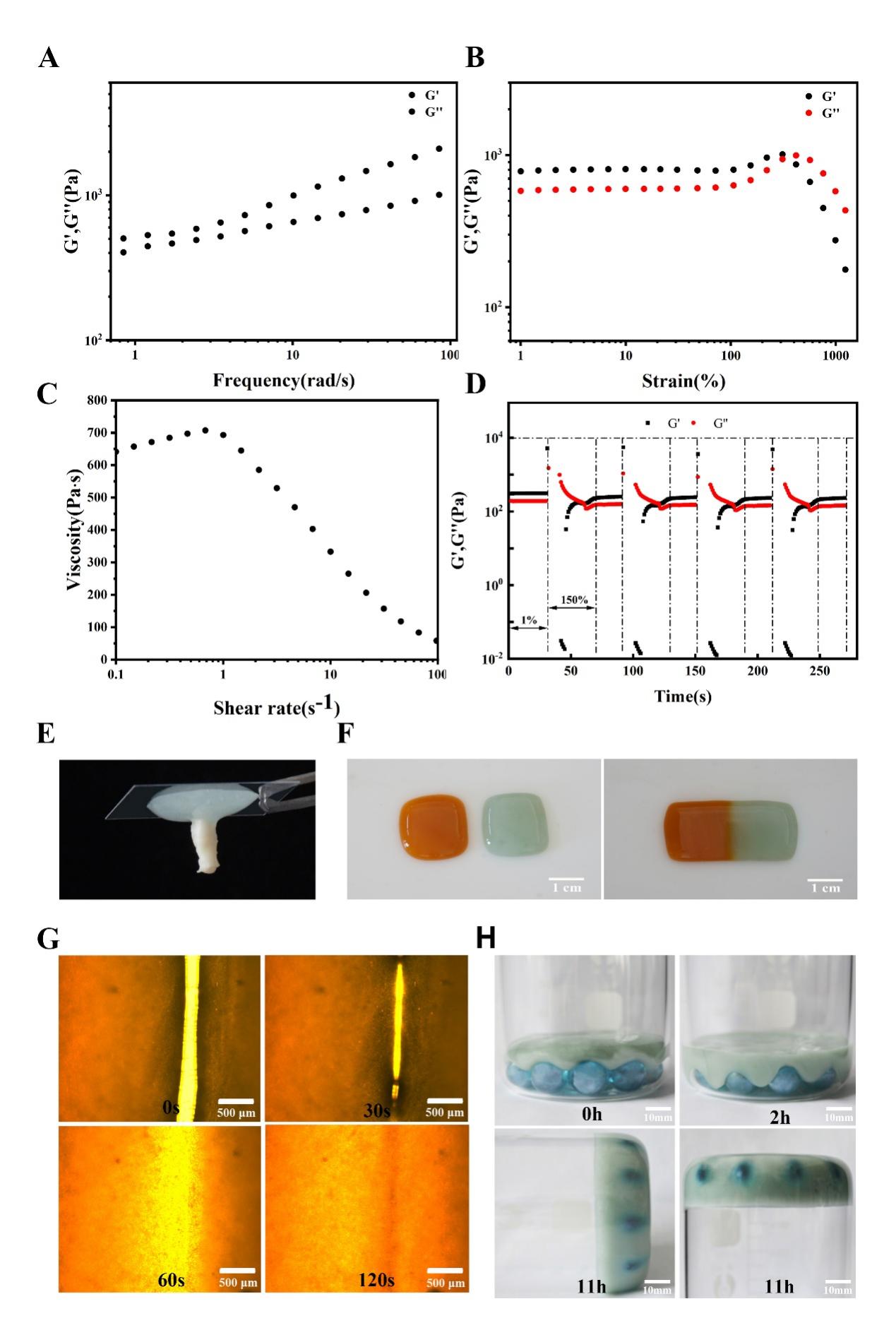


**Fig. S2 Mechanical Characteristics of COCu Hydrogel.**

(A) Strain sweep of COCu hydrogels; (B) Angular frequency sweep of COCu hydrogels; (C) Time sweep of COCu hydrogel with alternating strains of 1% (recovery) and 150% (break); (D) Viscosity graph of COCu hydrogel at different shear rates; (E) Spinal cord adhesion experiment of COCu hydrogel; (F) Macroscopic performance of self-healing function in COCu hydrogel; (G) Microscopic demonstration of self-healing function in COCu hydrogel; (H) Auto-filling function of COCu hydrogel.


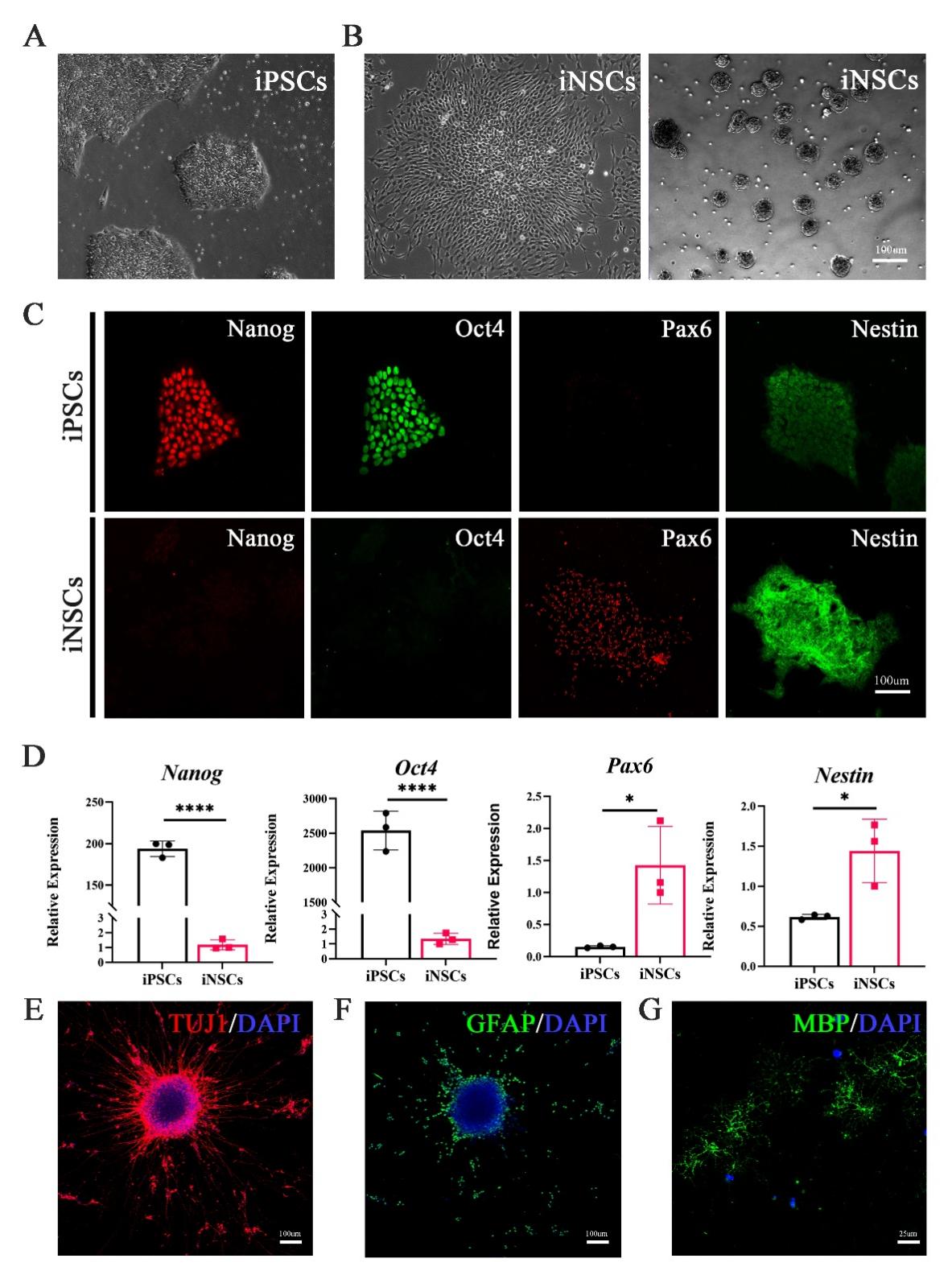


**Fig. S3 Validation of iPSCs and Induction to iNSCs Fate.**

(A) Microscope images of iPSCs; (B) Microscope images of iNSCs under adherent and suspension culture conditions, Scale bar, 100 μm; (C) Immunofluorescence staining of iPSC and iNSC. iPSC-specific markers were labeled with Nanog and OCT4, iNSC-specific markers were labeled with Pax6 and Nestin, Scale bar, 100 μm; (D) RT-PCR to detect the expression levels of iPSC-specific markers and iNSC-specific markers in iPSCs and iNSCs (n=3), * P < 0.05, **** P < 0.0001; (E-G) Immunofluorescence staining of iNSCs after 7 days of differentiation. Neurons, astrocytes, and oligodendrocytes were labeled with Tuj1, GFAP, and MBP, respectively. Scale bar, 100 μm and 25 μm.


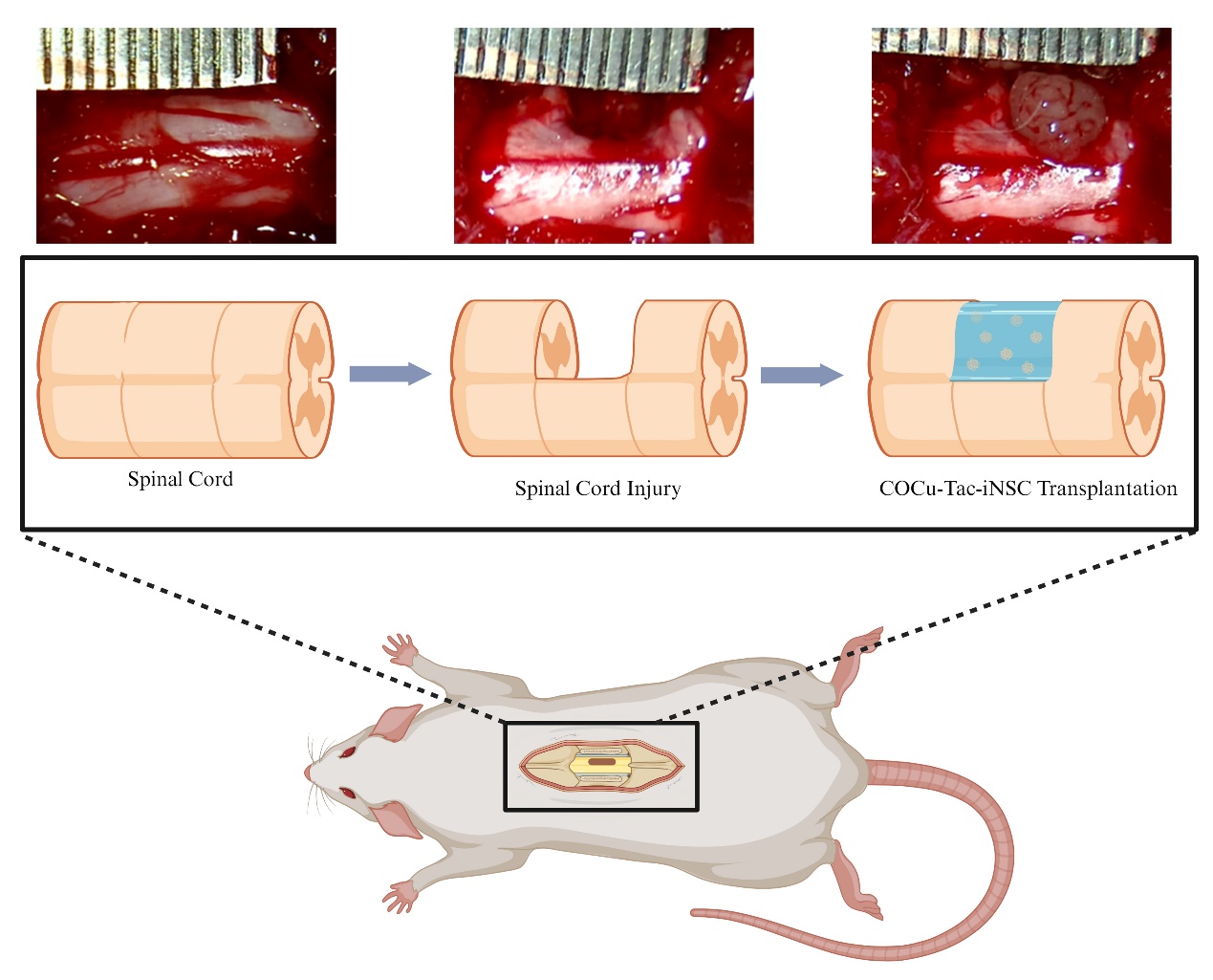
**Fig. S4 Intraoperative Photos and Schematics of Animal Surgery.**


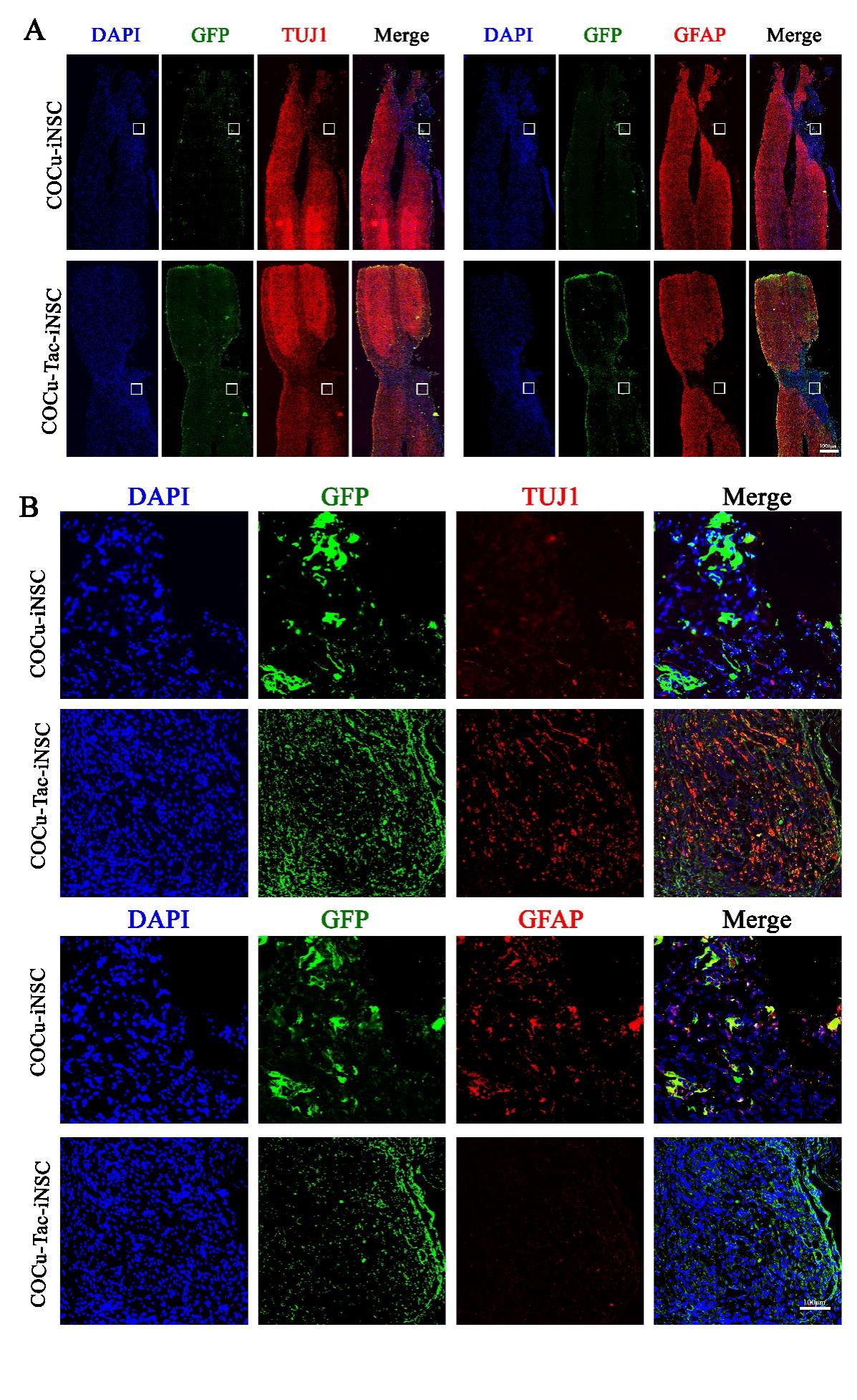


**Fig. S5 COCu-Tac Hydrogel Promotes the Survival and Neuronal Differentiation of Transplanted iNSCs.**

(A) Immunofluorescence staining of GFP+iNSCs in spinal cords 6 weeks after injury. iNSCs, neurons, and astrocytes were labeled with GFP, TUJ1, and GFAP, respectively. (B) Enlarged pictures of (A), Scale bar, 1000 μm for original pictures and 100 μm for enlarged pictures.


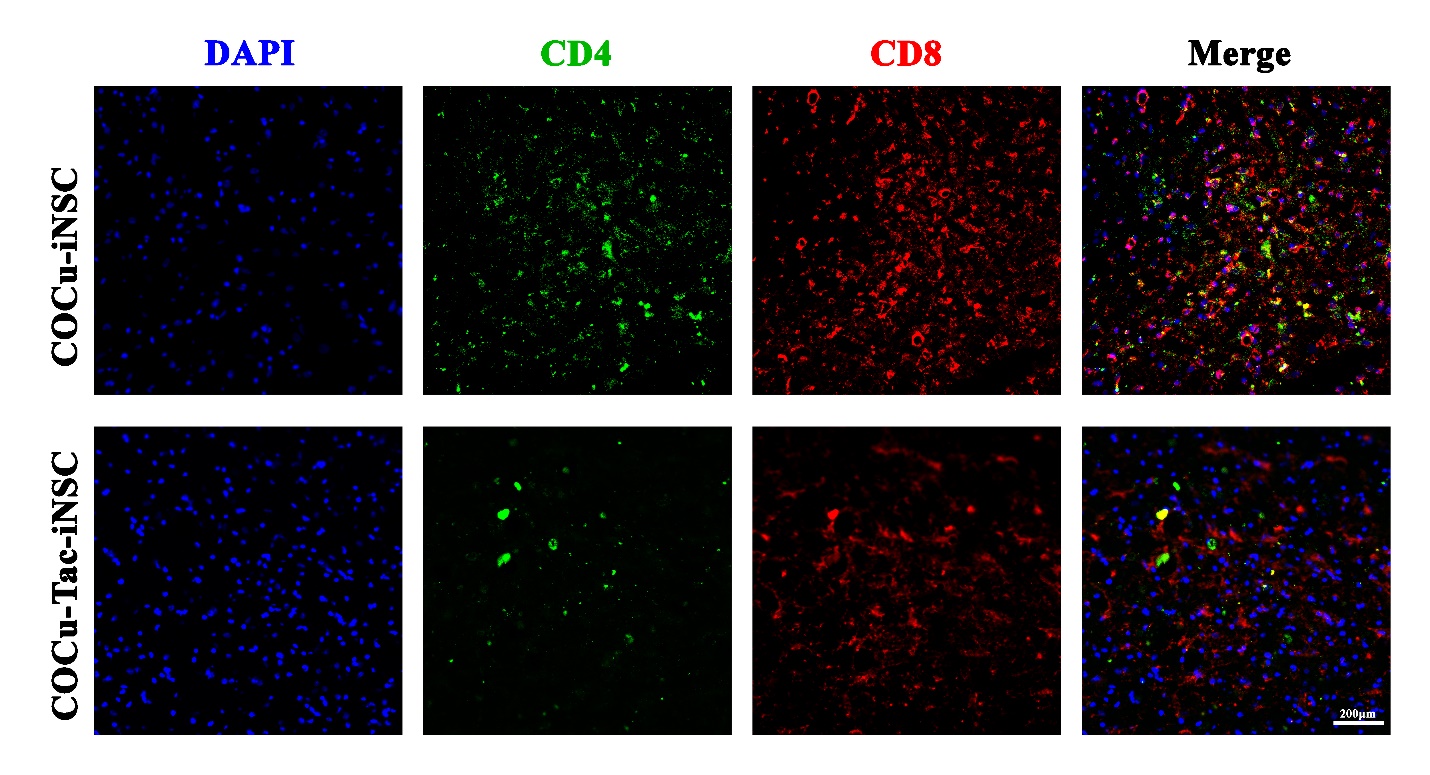
 **Fig. S6. Invasion of CD4^+^ T cells and CD8^+^ T cells 7 Days Post-surgery.**

Immunofluorescence staining of CD4^+^ T cells and CD8^+^ T cells in spinal cords 7 days after injury.


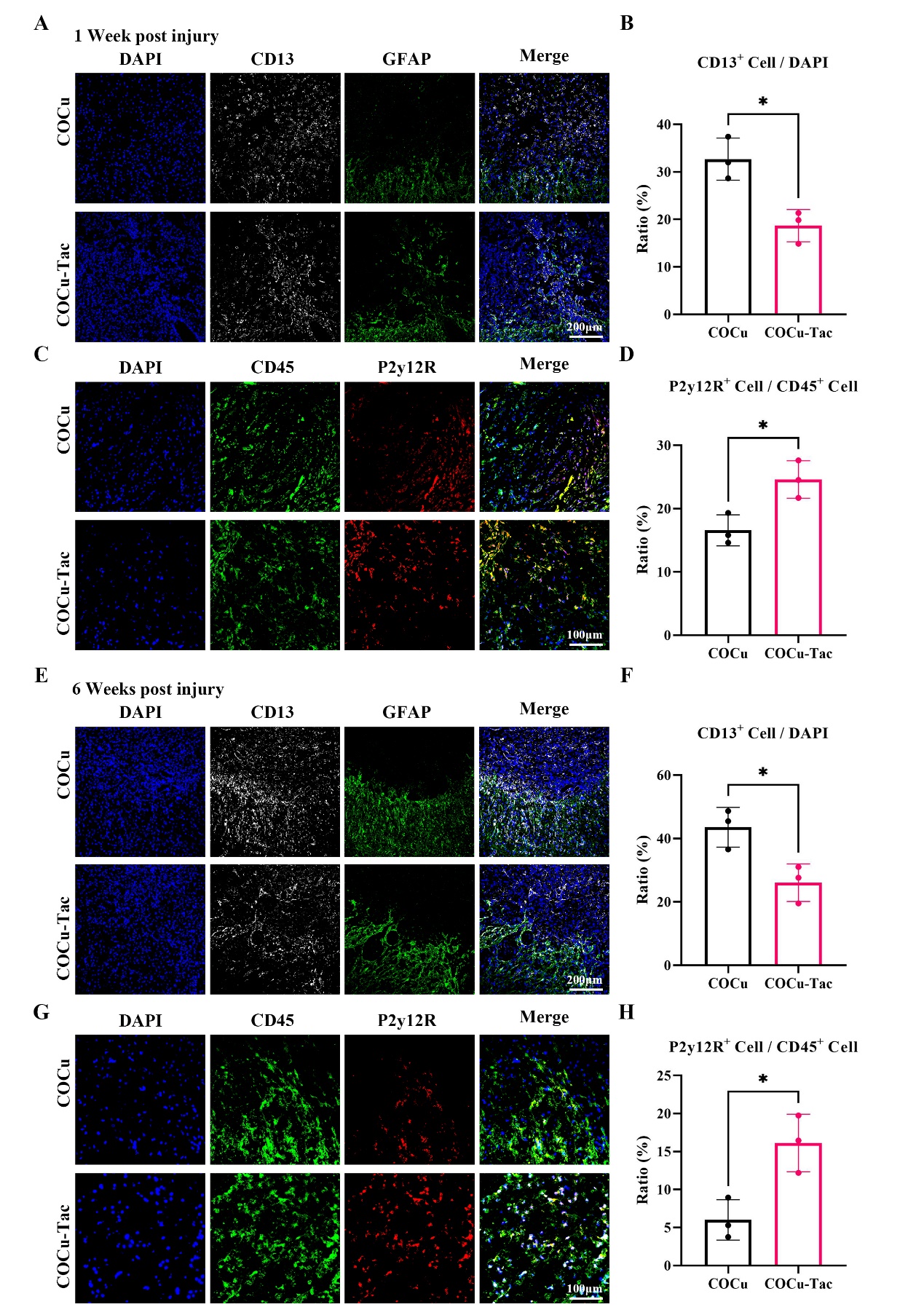


**Fig. S7. Invasion of Foreign Body Reaction Post-surgery.**

(A) Immunofluorescence of staining of CD13 and GFAP in spinal cords 1 week after injury, Scale bar, 200 μm; (B) Quantification of the average level of colocalization between CD13 and DAPI in (A) (n=3); (C) Immunofluorescence of staining of CD45 and P2y12R in spinal cords 1 week after injury, Scale bar, 100 μm; (D) Quantification of the ratio of P2y12R+ cells and CD45+ cellsI in (C) (n=3); (E) Immunofluorescence of staining of CD13 and GFAP in spinal cords 6 week after injury, Scale bar, 200 μm; (F) Quantification of the average level of colocalization between CD13 and DAPI in (E) (n=3); (G) Immunofluorescence of staining of CD45 and P2y12R in spinal cords 6 week after injury, Scale bar, 100 μm; (H) Quantification of the ratio of P2y12R+ cells and CD45+ cellsI in (G) (n=3), Scale bar, 10 μm * P < 0.05.


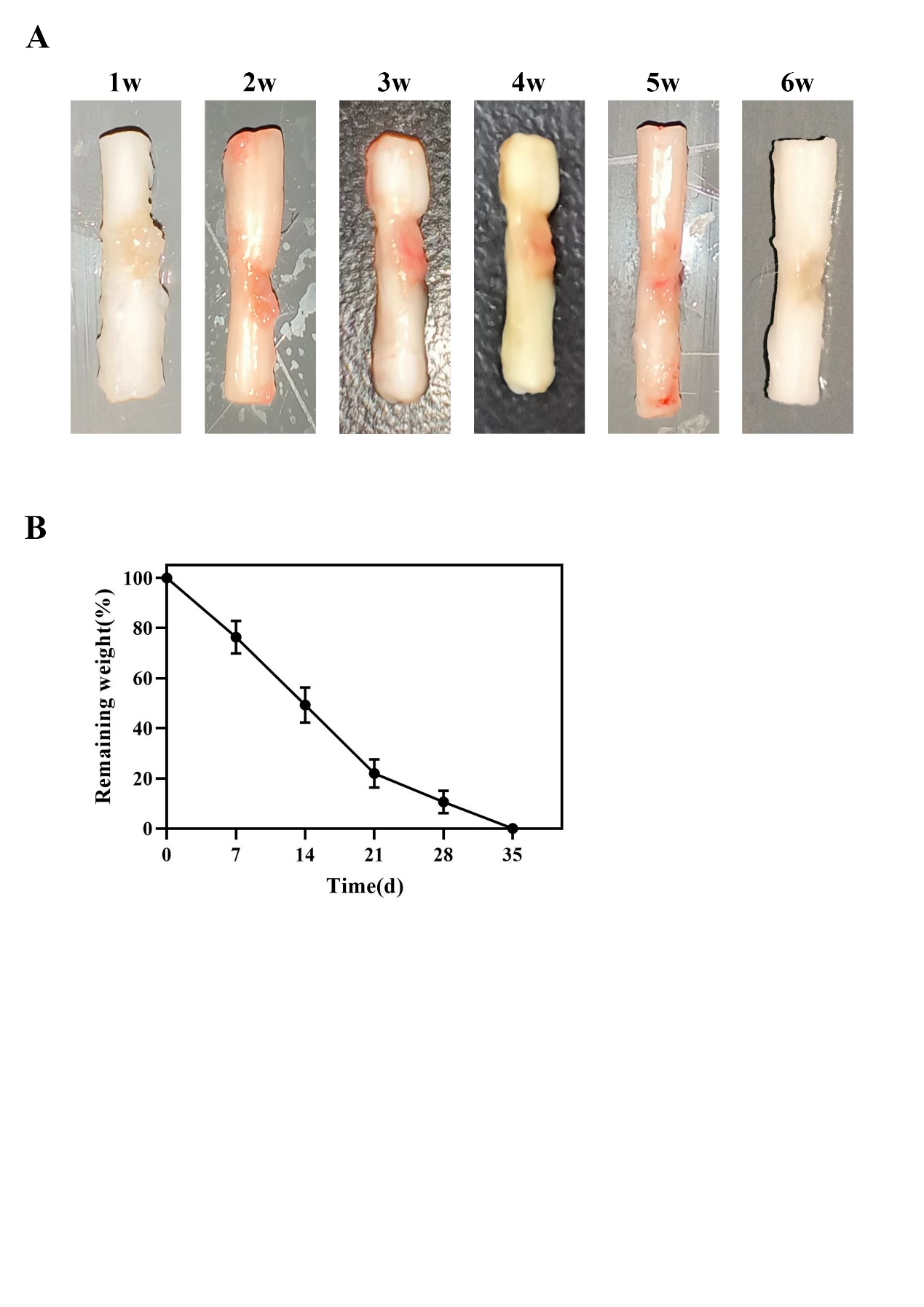


**Fig. S8. In Vivo Degradation of COCu-Tac Hydrogel.**

(A) Gross images of the spinal cord after COCu-Tac hydrogel implantation. (B) In vivo degradation curves of COCu-Tac Hydrogel.


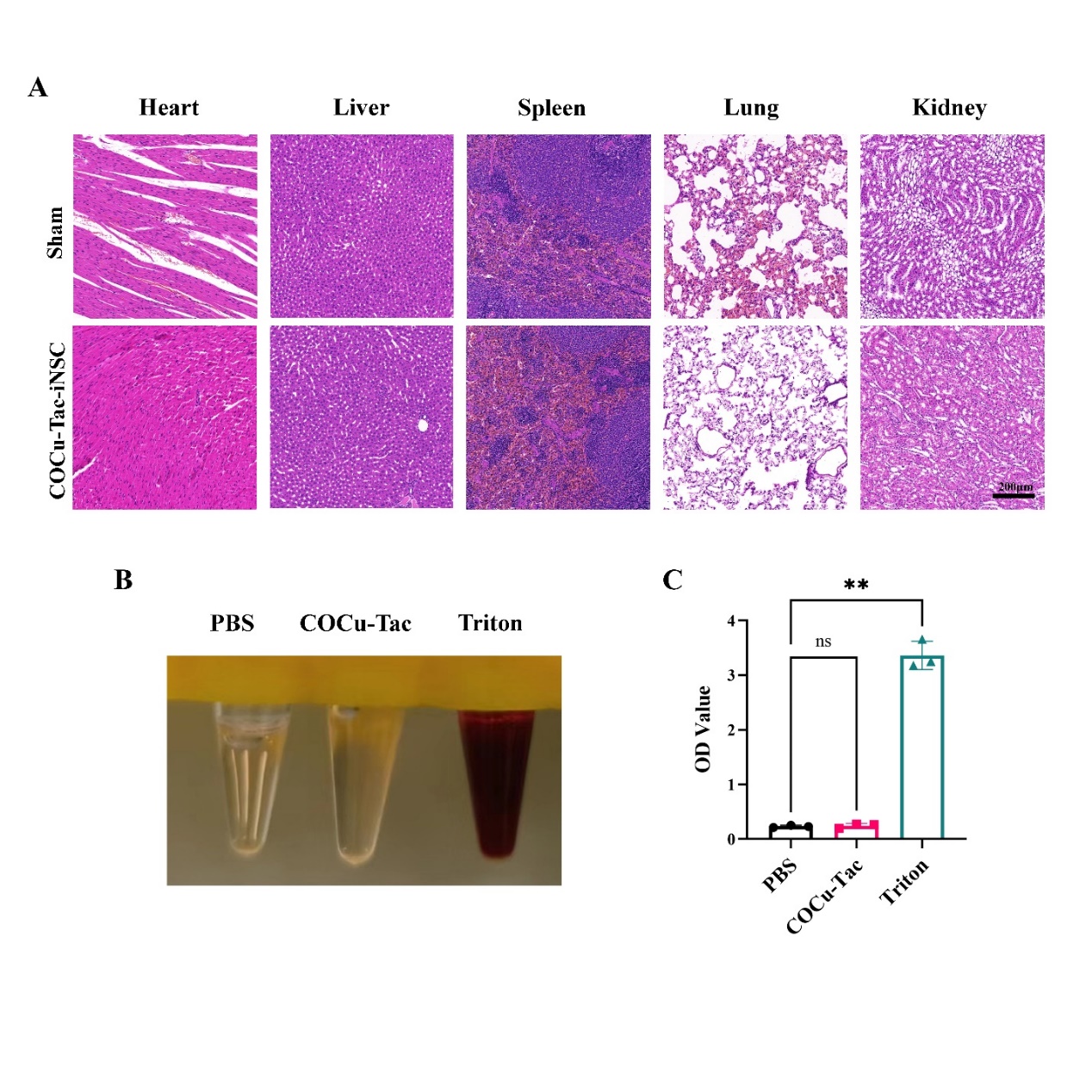
**Fig. S9. Biocompatibility of COCu-Tac Hydrogel.**

(A) HE staining of the heart, liver, spleen, lungs, and kidneys in rats from the Sham group and COCu-Tac-iNSC group six weeks post-surgery; (B) Hemolysis assay of COCu-Tac hydrogel, PSB and Triton served as negative and positive controls, respectively; (C) Quantitative detection of absorbance in each group of (B) (n=3), **P < 0.01, ns means no significance..

Table S1.

PCR Primer

| Primer Name | Species | 5' -3' | Sequence |
| --- | --- | --- | --- |
| Nestin | Human | Forward | AGCTGGCGCACCTCAAGATG |
| Nestin | Human | Reverse | AGGGAAGTTGGGCTCAGGAC |
| Pax6 | Human | Forward | GTACTGAATGACTCAACTGCTCGG |
| Pax6 | Human | Reverse | CTTTAGAAGGAAGCGACACTCTGC |
| Nanog | Human | Forward | AGTATGGTTGGAGCCTAATCAGCG |
| Nanog | Human | Reverse | ATCCTGGCTAACACAGTGAAACCC |
| Oct4 | Human | Forward | ATGCATTCAAACTGAGGTGCCTGC |
| Oct4 | Human | Reverse | CCCTTTGTGTTCCCAATTCCTTCC |
| Nefh | Rat | Forward | GGAGTGGTTCCGAGTGAGATTG |
| Nefh | Rat | Reverse | CCTTGGTGCTTTTCAGTGCCT |
| Tubb3 | Rat | Forward | CCCGTTTTAGCCACCTTTGTATT |
| Tubb3 | Rat | Reverse | CCCTCCAAATATAAACACAACCC |
| Gap43 | Rat | Forward | GCACATCGGCTTGTTTAGGCT |
| Gap43 | Rat | Reverse | GGAGGGAGATGGCTCTGCTACT |
| Syn1 | Rat | Forward | CTTCCTGGTTGGGGACTACTCCTC |
| Syn1 | Rat | Reverse | GCGAACACGGCTGTAGCCAGAAAG |
| Dlg4 | Rat | Forward | AAGATGAAGACACGCCCCCTC |
| Dlg4 | Rat | Reverse | TGTTCCATTCACCTGCAACTCA |
| Gfap | Rat | Forward | AGCTGTTGTTCTCCAAGCCAAG |
| Gfap | Rat | Reverse | CACAACGTGTGTCAGTTTCCCA |
